# The effects of CEP-37440, an inhibitor of focal adhesion kinase, in vitro and in vivo on inflammatory breast cancer cells

**DOI:** 10.1186/s13058-016-0694-4

**Published:** 2016-03-24

**Authors:** Israa Salem, Manal Alsalahi, Inna Chervoneva, Lucy D. Aburto, Sankar Addya, Gregory R. Ott, Bruce A. Ruggeri, Massimo Cristofanilli, Sandra V. Fernandez

**Affiliations:** Department of Medical Oncology, Thomas Jefferson University, Philadelphia, PA USA; Division of Biostatistics, Department of Pharmacology and Experimental Therapeutics, Thomas Jefferson University, Philadelphia, PA USA; Cancer Genomics Facility, Kimmel Cancer Center, Thomas Jefferson University, Philadelphia, PA USA; Teva Branded Pharmaceutical Products R&D, West Chester, PA USA; Present address: Department of Medicine - Hematology and Oncology, Robert H. Curie, Comprehensive Cancer Center, Northwestern University, Chicago, IL USA; Present address: Incyte Pharmaceuticals, Wilmington, DE USA

**Keywords:** CEP-37440, FAK1, ALK, IBC, Inflammatory breast cancer, Triple-negative breast cancer, TNBC

## Abstract

**Background:**

Inflammatory breast cancer (IBC) is an aggressive type of advanced breast cancer with a poor prognosis. We recently found that focal adhesion kinase 1 (FAK1) is upregulated and phosphorylated (active) in IBC. In this study, we investigated the effect of CEP-37440, a dual inhibitor of FAK1 and anaplastic lymphoma kinase (ALK), using human IBC cell lines and preclinical models of IBC.

**Methods:**

Cell proliferation assays were performed in the presence of several concentrations of CEP-37440 using IBC and triple-negative breast cancer non-IBC cell lines. In vitro, we studied the expression of total FAK1, phospho-FAK1 (Tyr 397), total ALK and phospho-ALK (Tyr 1604). In vivo*,* we tested CEP-37440 using FC-IBC02, SUM149, and SUM190 IBC xenograft mouse models.

**Results:**

CEP-37440 at low concentration decreased the proliferation of the IBC cell lines FC-IBC02, SUM190, and KPL4, while not affecting the proliferation of normal breast epithelial cells. At higher concentration, CEP-37440 was also able to inhibit the proliferation of the IBC cell line MDA-IBC03 and the triple-negative non-IBC cell lines MDA-MB-231 and MDA-MB-468; the IBC cell line SUM149 showed a slight response to the drug. CEP-37440 decreased the cell proliferation of FC-IBC02, SUM190, and KPL4 by blocking the autophosphorylation kinase activity of FAK1 (Tyr 397). None of the cells evaluated expressed ALK. In vivo, after 7 weeks of CEP-37440 treatment, the SUM190, FC-IBC02, and SUM149 breast tumor xenografts were smaller in mice treated with 55 mg/kg bid CEP-37440 compared to the controls; the tumor growth inhibition (TGI) was 79.7 %, 33 %, and 23 %, respectively. None of the FC-IBC02 breast xenografts mice treated with CEP-37440 developed brain metastasis while 20 % of the mice in the control group developed brain metastasis. Expression array analyses in FC-IBC02 cells showed that CEP-37440 affects the expression of genes related to apoptosis, interferon signaling, and cytokines.

**Conclusions:**

CEP-37440 is effective against some IBC cells that express phospho-FAK1 (Tyr 397), and its antiproliferative activity is related to its ability to decrease phospho-FAK1. Our results suggest that combinational therapies could be more effective than using CEP-37440 as a single agent.

**Electronic supplementary material:**

The online version of this article (doi:10.1186/s13058-016-0694-4) contains supplementary material, which is available to authorized users.

## Background

Inflammatory breast cancer (IBC) is a very aggressive type of advanced breast cancer with a poor prognosis. IBC occurs typically in patients under the age of 50 and is often misdiagnosed as an infection since it does not present as a lump [1]. The clinical symptoms of IBC involve the rapid onset of changes in the skin overlying the breast, including edema, redness, and swelling exhibiting a wrinkled, orange peel-like appearance of the skin known as peau d’orange [2]. This peculiar presentation is associated with the invasion of aggregates of tumor cells, defined as tumor emboli, into the dermal lymphatics, where they obstruct the lymph channels [3, 4]. Although IBC currently accounts for only 2–6 % of all breast cancer cases in the United States and up to 20 % of all breast cancers globally [1, 5–7], its incidence is dramatically increasing [2, 4]. Furthermore, due to its propensity to rapidly metastasize, IBC is responsible for a disproportionate number (15 %) of breast cancer-related deaths [7–9]. IBC is either stage III or IV disease, depending on whether cancer cells have spread only to nearby lymph nodes or to other tissues as well. At the time of diagnosis, most IBC patients have lymph node metastases, and approximately 30 % have distant metastases in brain, bones, visceral organs, and soft tissue [1]. Currently, there is no adequate adjuvant therapy to reduce the risk of recurrence and mortality in IBC patients.

We recently found that focal adhesion kinase 1 (FAK1 or PTK2) is amplified, upregulated and phosphorylated (active) in IBC [10]. FAK1 is a nonreceptor tyrosine kinase that localizes to areas termed focal adhesions where the cell membrane attaches to the extracellular matrix. FAK1 activation relies upon autophosphorylation of the Tyr 397 site that is found in the N-terminal domain; FAK1 Tyr 397 binds various signaling proteins, including Src, PI-3 kinase, and Grb-7. FAK1 also binds epidermal growth factor receptor (EGFR), vascular epidermal growth factor receptor (VEGFR), p53, and other molecules that are critical for tumor growth and progression [11]. It controls various cellular pathways, including proliferation, viability, and survival, and its overexpression has been linked to anoikis resistance [12–15]. The activation and phosphorylation of FAK1 stimulated by many forms of oncogenic transformation provide a plausible mechanism for the anchorage-independent growth of cancer cells. FAK1 overexpression correlates negatively with patient outcome and is associated with increased cell migration, invasion, and metastasis; elevated FAK1 expression has been reported in multiple human epithelial tumors [14, 16–20].

CEP-37440 is a potent ATP-competitive, highly kinase selective, and orally active inhibitor of FAK1 (enzymatic IC_50_: 2.3 nM, cellular IC_50_: 88 nM) and anaplastic lymphoma kinase (ALK) (enzymatic IC_50_: 3.5 nM, cellular IC_50_: 40 nM, cellular IC_50_ in 75 % human plasma: 120 nM) [21]. In addition to a favorable metabolic stability and pharmacokinetic profile preclinically, CEP-37440 is also a brain penetrant [21]. In this study, we investigated the effect of CEP-37440 using human IBC cells and preclinical models of IBC. We found that CEP-37440 was able to inhibit the proliferation of certain IBC cells by decreasing the levels of phospho-FAK1 (Tyr 397); none of the cells expressed ALK. Studies using IBC xenograft models showed that CEP-37440 also effectively reduces the growth of the primary tumor xenografts and inhibits the development of brain metastases in mice.

## Methods

### Cell lines

The cell lines used are described in Table 1. The SUM149 and SUM190 cell lines were purchased from Asterand Inc. (Detroit, MI, USA). The MDA-IBC03 cells were obtained from W.A. Woodward, and KPL4 cells were obtained from N.T. Ueno, The University of Texas MD Anderson Cancer Center, TX, USA. All other cell lines, MDA-MB-231, MDA-MB-468, MCF-10A, and MCF-12A, were purchased from American Type Culture Collection (ATCC; Manassas, VA, USA). The new model of IBC, designated as FC-IBC02, was previously developed in our laboratory, through the isolation of tumor cells from the pleural effusion of an IBC patient, as described elsewhere [10].

**Table 1:** Sensitivity of the cell lines to CEP-37440

Cell line	Type	Subtype			GI_50_ (nM)	Reference
		ESR	PgR	ErbB2		
FC-IBC02	IBC	Neg	Neg	Neg	91	[10]
KPL4	IBC	Neg	Neg	Post	~900	[41]
SUM190	IBC	Neg	Neg	Post	890	[42]
MDA-IBC03	IBC	Neg	Neg	Post	1860	[43]
SUM149	IBC	Neg	Neg	Neg	>3000	[42]
MCF-12A	Normal-like breast epithelial cells	Neg	Neg	Neg	1516	[44]
MCA-10A	Normal-like breast epithelial cells	Neg	Neg	Neg	1700	[45]
MDA-MB-231	BC (non-IBC)	Neg	Neg	Neg	1930	[46]
MDA-MB-468	BC (non-IBC)	Neg	Neg	Neg	1275	[46]

Inflammatory breast cancer (IBC), breast cancer (BC) non-IBC, and normal-like breast epithelial cell lines were grown in the presence of different concentrations of CEP-37440 (cell proliferation assays). The GI_50_ indicate the CEP-37440 concentration required to reduce growth rates to 50 % of the maximum rate. The GI_50_ was calculated for each cell line at t = 144 h of CEP-37440 treatment using sigmoidal dose response curve (variable slope) in GraphPad Prism (GraphPad Software Inc., La Jolla, CA, USA)

*ESR* estrogen receptor, *PgR* progesterone receptor, *ErbB2* epidermal growth factor receptor 2

### Reagents

CEP-37440 was synthesized and provided by Teva Branded Pharmaceutical Products R&D, West Chester, PA, USA. CEP-37440 has modest plasma protein binding, high intrinsic solubility, reduced lipophilicity, favorable microsomal metabolic stability across species, reduced capacity for drug–drug interaction, and possesses favorable oral bioavailability and a lower clearance rate in vivo across multiple species [21]. For in vitro assays, CEP-37440 free base was dissolved in dimethyl sulfoxide (DMSO) at concentration of 4 mM. For in vivo studies, CEP-37440 tri-hydrochloride di-hydrate salt was used and the drug was dissolved in distilled water. The media for the different cell lines are described in (Additional file 12).

### Total FAK1 and phospho-FAK1 (Tyr 397) assays

Cells were grown to 80 % confluence in 100 mm in diameter Petri dishes. Cells were collected in cold phosphate-buffered saline (PBS) by scraping from culture plates, centrifuged, and washed twice with cold PBS; proteins were extracted with 500 μl of cell extraction buffer (Invitrogen, Life Technologies, Carlsbad, CA, USA) containing a mixture of protease inhibitors (Protease Inhibitors Cocktail, Sigma-Aldrich, St. Louis, MO, USA) and 1 mM phenylmethanesulfonyl fluoride (PMSF). After extraction for 30 min on ice, the extracts were centrifuged at 14,000 × g for 10 min at 4 °C, and the supernatant was transferred to a fresh microcentrifuge tube and kept at −80 °C until use. The protein concentration in the cell extracts was determined using the BCA protein assay kit (Pierce, Rockford, IL, USA). Total FAK1 was studied in the cell protein extracts using FAK (Total) enzyme-linked immunosorbent assay (ELISA) kits (Invitrogen, Frederick, MD, USA); cell extracts were diluted 1:40 in dilution buffer as was recommended by the manufacturer; total FAK1 was expressed as ng total FAK1/μg protein. To study phospho-FAK1, FAK (pY397) ELISA kits (Invitrogen, Life Technologies, Carlsbad, CA, USA) were used and cell extracts were diluted 1:5 in dilution buffer; phospho-FAK1 (Y397) was expressed as units of phospho-FAK1/μg protein.

### Total ALK and phospho-ALK (Tyr 1604) assays

Total ALK and phospho-ALK (Tyr 1604) were studied using PathScan Total ALK Sandwich ELISA and PathScan Phospho-ALK (Tyr 1604) Sandwich ELISA kits, respectively (Cell Signaling Technology, Inc., Danvers, MA, USA); protein extracts from Karpas 299 cells, a human T cell lymphoma cell line, were used as positive control.

### Cell proliferation assays

For FC-IBC02, SUM190, MDA-IBC03, SUM149, MDA-MB-231, MDA-MB-468, MCF-10A, and MCF-12A, a total of 2000 cells were plated per well in a 96-well plate, except for KPL4 in which 1000 cells were plated. After the cells were plated, they were allowed to attach overnight and then treated with CEP-37440 during several time points (0 h, 24 h, 48 h, 72 h, 96 h, 120 h, 144 h, 168 h, and 192 h). Several CEP-37440 concentrations were evaluated (0, 3, 10, 30,100, 300, 1000, 2000, and 3000 nM), and each concentration was tested in quadruplicate; as controls, cells growing in regular media (without CEP-37440) and media with 0.075 % DMSO (vehicle) were included. After CEP-37440 treatments, the living cells were quantified using the CellTiter 96 Aqueous One Solution Cell Proliferation Assay kit (Promega, Madison, WI, USA) following the manufacturer’s instructions. Briefly, the media with the CEP-37440 was removed, and 100 μl of fresh media was added to each well; 20 μl 3-(4,5-dimethylthiazol-2-yl)-5-(3-carboxymethoxyphenyl)-2-(4-sulfophenyl)-2H-tetrazolium (MTS) compound was added and the cells were incubated at 37 °C in a cell incubator for 4 h. The absorbance at 490 nm and 630 nm (reference wavelength) was measured with a 96-well microplate reader (iMark™, Bio-Rad Laboratories Inc., Hercules, CA, USA).

### Gene expression profiling

FC-IBC02 cells were treated with 1000 nM CEP-37440 for 48 h and RNA was isolated. Affymetrix Genechip® Human Gene 1.0 ST array (Affymetrix Inc., Santa Clara, CA, USA) were used for gene expression studies (Additional file 12). Data analyses were performed using GeneSpring software 13.1 (Agilent Technologies, Inc., Santa Clara, CA, USA). The criteria for differentially expressed genes were set at ≥ 2.0-fold changes. Statistical analysis was performed to compare two groups using *t* test unpaired with a *p* value less than or equal to 0.05. A heat map was generated from the differentially expressed gene list. The list of differentially expressed genes was loaded into Ingenuity Pathway Analysis (IPA) 8.0 software (http://www.ingenuity.com) to perform biological network and functional analyses.

### In vivo studies using SCID mice

Studies were approved by the Institutional Animal Care Committee at Thomas Jefferson University. A total of 10^6^ cells were suspended in 100 μl PBS, mixed with 100 μl Matrigel (BD Biosciences, Bedford, MA, USA), and injected into the fourth left inguinal mammary fat pad of severe combined immune-deficient (SCID) mice. The animals were palpated daily for detection of tumor development and, once the breast tumor xenografts reached approximately 50–100 mm^3^ (approximately 20–30 days postinjection), the mice were randomly allocated into groups. Two doses of CEP-37440 were tested for mice harboring FC-IBC02 or SUM149 breast tumor xenografts; the mice allocated to treatment received either 30 mg/kg twice a day (bid) or 55 mg/kg bid by oral gavage in a volume of 100 μl, 5 days/week for 35–40 days. For mice harboring SUM190 breast tumor xenografts, only the higher CEP-37440 dose (55 mg/kg bid) was tested. The CEP-37440 doses were chosen based on preliminary experiments in order to achieve an optimal plasma concentration–response relationship [21]. Breast tumors were measured using a vernier caliper, and tumor volumes were calculated using the following equation: V = [(L1 + L2)/2] × L1 × L2 × 0.526 where L1 and L2 are the length and width of the tumor. After 40 days of treatment or when the primary tumor reached a volume of approximately 1 cm^3^, the animals were euthanized by carbon dioxide (CO_2_) inhalation. Breast tumors and other organs (lungs, heart, liver, spleen, brain, ovaries, kidneys, and lymph nodes) were removed, fixed in 10 % neutral-buffered formalin and paraffin-embedded for histological examination (Additional file 12).

### Statistical analyses

For the analyses of the cell proliferation data, the log-transformed response measures (Abs 490 nm and Abs 630 nm) were modeled using the linear mixed effects (LME) model adjusting for correlations between repeated measures over time. The fixed effects included the ten concentrations and linear time trends. For the analyses of in vivo tumor growth data, the log-transformed tumor volumes were modeled using LME models adjusting for correlations between repeated measures from the same animal. The fixed effects included the control group and treatment groups (30 mg/kg CEP-37440, and 55 mg/kg CEP-37440), and linear and quadratic time trends. The LME models included either only linear terms or both linear and quadratic terms as appropriate for specific time-dependent trends. Percent tumor growth inhibition (% TGI) was calculated as follows: 100 × [(tumor volume of the control group at the end of treatment − tumor volume of the treated group at the end of the treatment)/tumor volume of the control group at the end of the treatment] = 100 × (1-exp (mean difference in log volumes at the end of the treatment), where the mean difference in log volumes at the end of the treatment was estimated from the fitted LME models. The data were analyzed using R package ‘nlme’ (The R Foundation for Statistical Computing http://www.R-project.org).

## Results

### FAK1 is phosphorylated in IBC cell lines

**Fig. 1:**
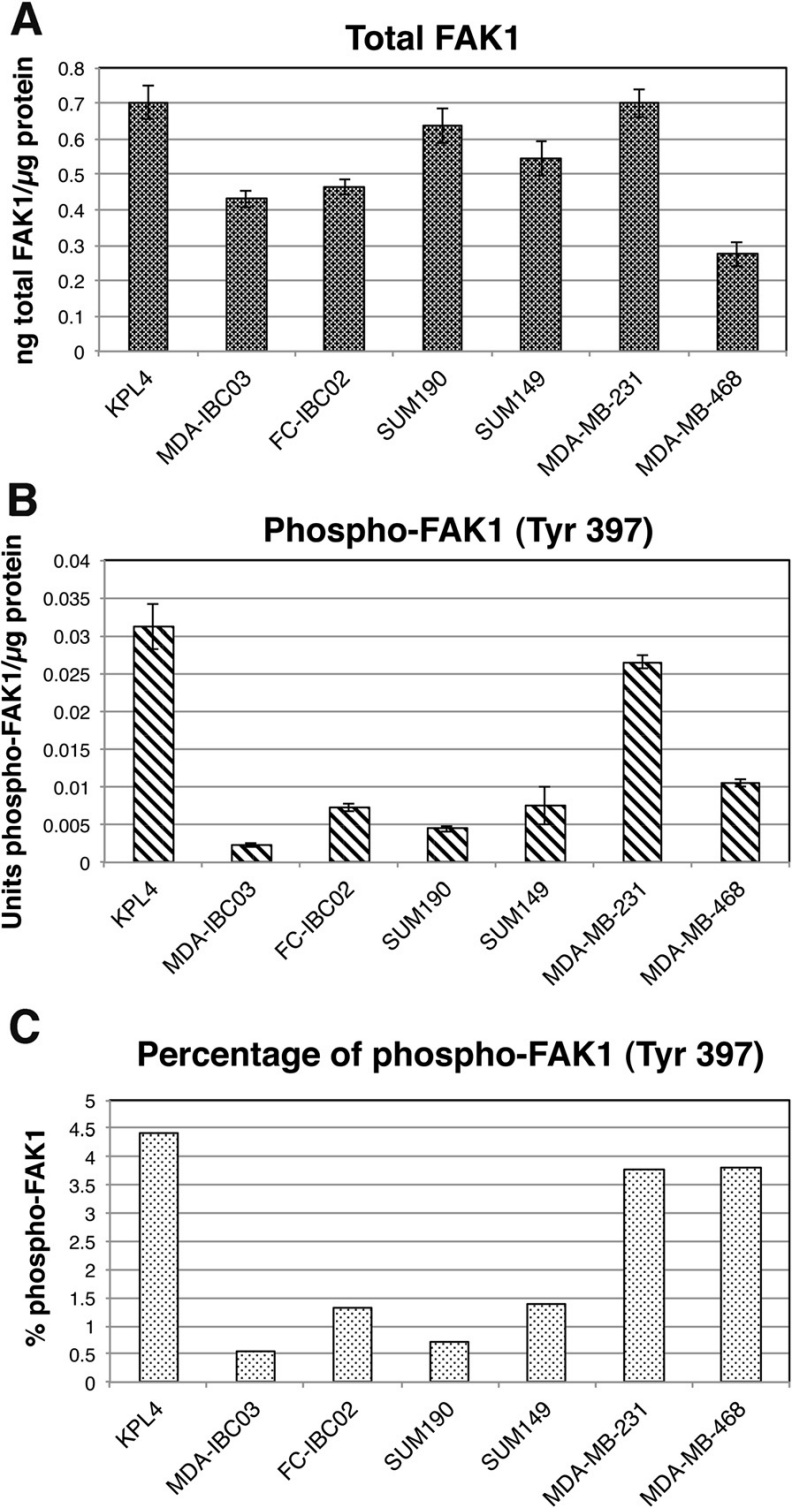
Total and phosphorylated FAK1 in IBC and non-IBC cell lines. **a** Total FAK1; **b** phospho-FAK1 (Tyr 397); **c** percentage of phospho-FAK1 compared to total FAK1 in different cell lines ([phospho-FAK1/total FAK1] × 100). Standard errors (SE) are indicated in each case. *FAK1* focal adhesion kinase 1, *IBC* inflammatory breast cancer

Using ELISA, total FAK1 and phospho-FAK1 (Tyr 397) were studied in the IBC cell lines KPL4, MDA-IBC03, FC-IBC02, SUM190, and SUM149, and in the non-IBC triple-negative cell lines MDA-MB-231 and MDA-MB-468. All of the cell lines expressed high levels of FAK1 (between 0.28 ng to 0.7 ng FAK1/μg protein), and no differences were observed between IBC and non-IBC cell lines (Fig. 1a). However, the levels of phospho-FAK1 (Tyr 397) differed between the cell lines (Fig. 1b). The percentage of phosphorylated FAK1 (Tyr 397) in relation of total FAK1 was higher in KPL4, MDA-MB-231 and MDA-MB-468 (Fig. 1c); 4.4 % of total FAK1 was phosphorylated in KPL4, and approximately 3.6 % was phosphorylated MDA-MB-231 and MDA-MB-468. A lower percentage of FAK1 was phosphorylated in FC-IBC02 (1.4 %), SUM149 (1.4 %), SUM190 (0.66 %), and MDA-IBC03 (0.54 %) (Fig. 1c). We also studied the expression of FAK1 and phospho-FAK1 in the human normal-like breast epithelial cells MCF-10A and MCF-12A; these cells express FAK1 (approximately 0.5 ng total FAK1/μg protein and 0.26 ng total FAK1/μg protein, respectively) but they did not express phospho-FAK1 (Tyr 397).

### ALK was not expressed by IBC cell lines

The IBC cell lines FC-IBC02, SUM190, KPL4, SUM149 and MDA-IBC03 were tested for expression of ALK by ELISA. The human T cell lymphoma cell line Karpas 299 that carries the NPM (nucleophosmin)-ALK fusion gene was used as positive controls and these cells were strongly positive for total ALK and phospho-ALK (Tyr 1604). All of the IBC cell lines tested were negative for total ALK and phospho-ALK (Tyr 1604), and the non-IBC cell lines MDA-MB-231 and MDA-MB-468 were also negative for total ALK and phospho-ALK expression (data not shown).

### CEP-37440 specifically decreased the proliferation of certain IBC cells in a concentration-dependent manner

**Fig. 2:**
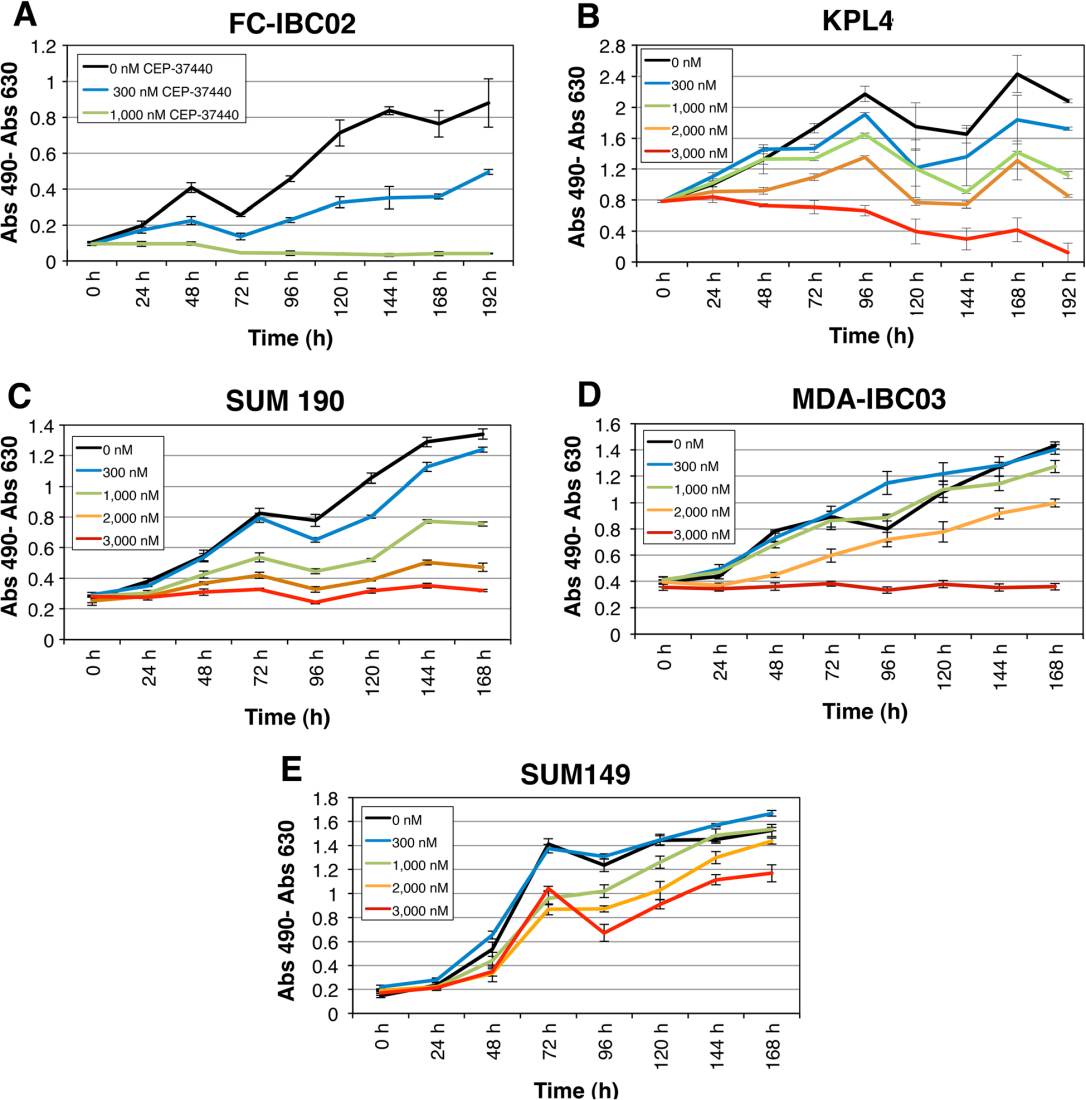
Effect of CEP-37440 on IBC cell lines proliferation. Several concentrations of CEP-37440 were tested on the IBC cell lines: **a** FC-IBC02, **b** KPL4, **c** SUM190, **d** MDA-IBC03, and **e** SUM149. CEP-37440 at low concentration (1000 nM) inhibited the proliferation of the IBC cells FC-IBC02, KPL4 and SUM190 but did not affect MDA-IBC03 and SUM149 proliferation. At higher concentration, 3000 nM CEP-37440, inhibited MDA-IBC03 proliferation. CEP-37440 did not inhibit SUM149 proliferation. Each point in the graphic represents the mean ± standard errors (SE) of four replicates. *IBC* inflammatory breast cancer

Cell proliferation rate was studied in the IBC cell lines in the presence of several concentrations of CEP-37440. The MTS assay that measures cellular respiration was used as a surrogate of cell proliferation. In the presence of 300 nM CEP-37440, FC-IBC02 significantly decreased in proliferation compared to the control without the drug, and 1000 nM CEP-37440 completely inhibited FC-IBC02 proliferation, killing these cells after 72 h of treatment (Fig. 2a). FC-IBC02 showed a positive linear growth rate when the cells were in the presence of lower concentrations of CEP-37440 (3 nM, 10 nM, 100 nM and 300 nM) (Additional file 1: Figure S1 and Additional file 2: Table S1); in contrast, the growth rates in the cells treated with high concentrations of CEP-37440 (1000 nM, 2000 nM and 3000 nM) were not significantly different from time 0 (Additional file 1: Figure S1 and Additional file 2: Table S1).

In KPL4 cells, treatment with 1000 nM CEP-37440 for 144 h reduced significantly the proliferation of cells compared to the control, and the growth rate was even lower in the presence of 2000 nM or 3000 nM CEP-37440 (Fig. 2b). The growth rate in the KPL4 cells treated with the 3000 nM CEP-37440 was the lowest (Additional file 3: Figure S2 and Additional file 4: Table S2).

**Fig. 3:**
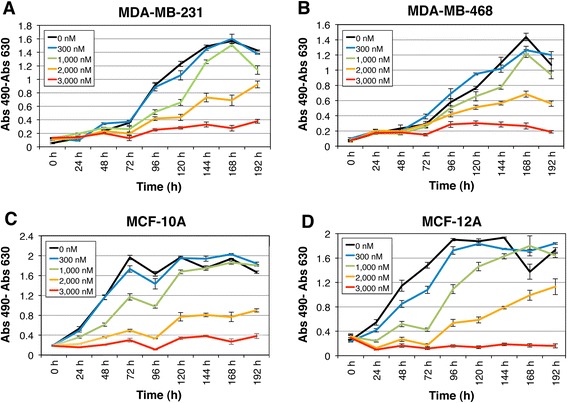
Effect of CEP-37440 on the proliferation of non-IBC breast tumor cells and normal breast epithelial cells. Several concentrations of CEP-37440 were tested on the cell lines: **a** MDA-MB-231, **b** MDA-MB-468, **c** MCF-10A and **d** MCF-12A. CEP-37440 at high concentration inhibited the proliferation of the triple-negative non-IBC cell lines MDA-MB-231 and MDA-MB-468 but, also decreased the proliferation of normal breast epithelial cells. A concentration of 3000 nM CEP-37440 inhibited MDA-MB-231 and MDA-MB-468 proliferation but also inhibited the proliferation of the normal-like breast epithelial cells MCF-10A and MCF-12A. Each point in the graphic represents the mean ± standard errors (SE) of four replicates. *IBC* inflammatory breast cancer

In SUM190 cells, treatment with 1000 nM CEP-37440 for 144 h reduced proliferation to approximately 60 % (Fig. 2c). The response measures corresponding to all concentrations except for 3000 nM grew over time and were significantly different from zero rates (Additional file 5: Figure S3 and Additional file 6: Table S3). The IBC cell line MDA-IBC03 only responded to a very high concentration of CEP-37440 (Fig. 2d), and SUM149 showed a slight response to high CEP-37440 concentrations (Fig. 2e). We also tested the effect of CEP-37440 on the triple-negative non-IBC cell lines MDA-MB-231 and MDA-MB-468 (Fig. 3a and b). Treatment with 2000 nM CEP-37440 for 144 h reduced MDA-MB-231 and MDA-MB-468 proliferation to approximately 46–54 % (Fig. 3a, 3b). We also studied the potential cytotoxic effects of CEP-37440 on normal breast epithelial cells, MCF-10A and MCF-12A (Fig. 3c and d). We found that 300 nM or 1000 nM CEP-37440 did not affect the proliferation of these cells; 2000 nM CEP-37440 decreased their proliferation to approximately 50 % and 3000 nM CEP-37440 inhibited their proliferation almost completely (Fig. 3c, 3d). The drug concentration required to reduce growth rates to 50 % (GI_50_) for each cell line are shown in Table 1.

In conclusion, CEP-37440 at low concentration specifically reduced the proliferation of three out of five IBC cell lines; FC-IBC02, KPL4 and SUM190 cells showed decreased proliferation in the presence of 1000 nM CEP-37440, and this same concentration did not significantly reduce the proliferation of normal breast epithelial cells. The CEP-37440 concentrations required to reduced the growth rate to 50 % (GI_50_) for each cell line are indicated in Table 1.  The sensitivity of these IBC cell lines to CEP-37440 was not related to the cell subtype since FC-IBC02 is a triple-negative cell line, and KPL4 and SUM190 are human epidermal growth factor receptor 2; (ErbB2)-positive (Table 1). MDA-IBC03 and the triple-negative non-IBC cell lines MDA-MB231 and MDA-MB-468 were less sensitive to CEP-37440, and the IBC cell line SUM149 was slightly affected by the drug.

### CEP-37440 decreases phospho-FAK1 (Tyr 397) and maintains its low level over time in FC-IBC02, SUM 190, and KPL4

**Fig. 4:**
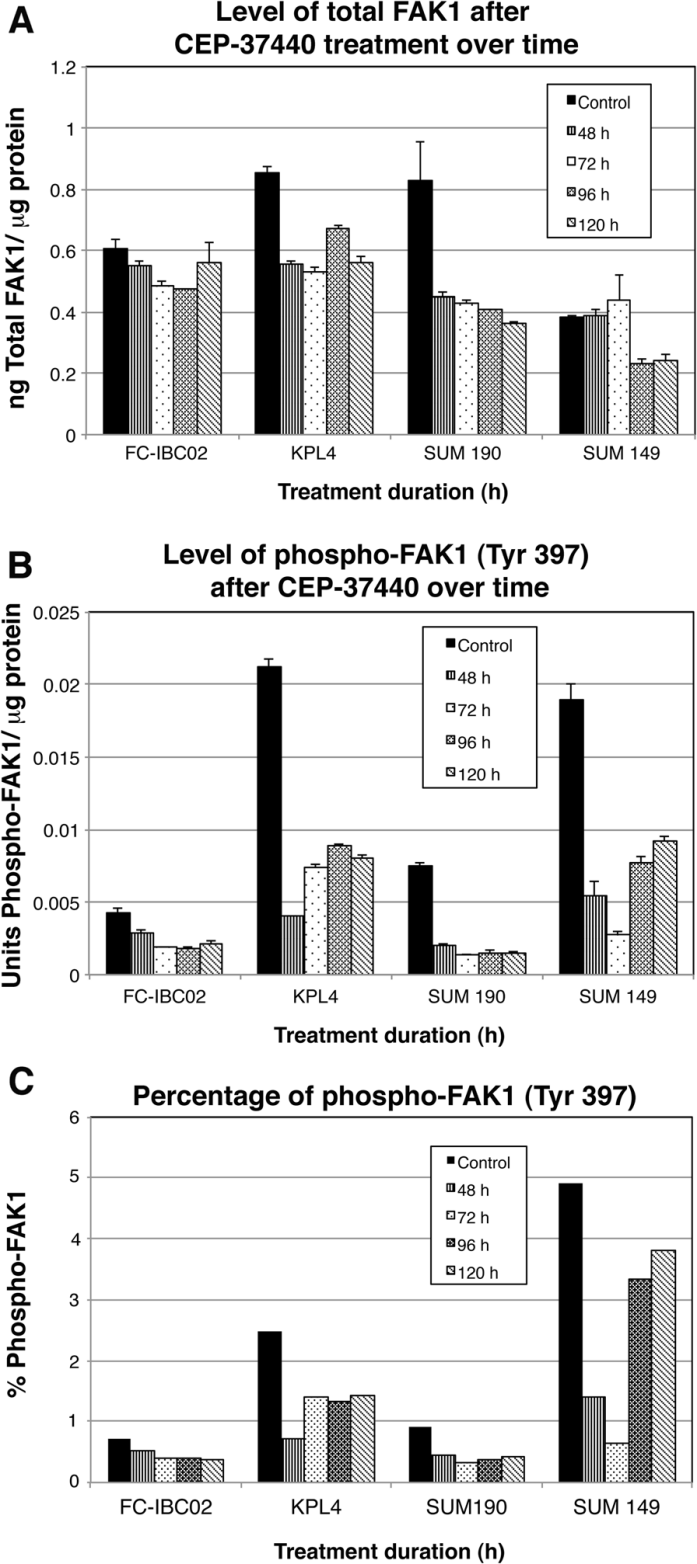
Total FAK1 and phospho-FAK1 in cells treated with 1000 nM CEP-37440 during time. **a** Total FAK1; **b** phospho- FAK1; **c** percentage of phospho-FAK1 ([phospho-FAK1/ total FAK1] x 100). Total and phospho-FAK1 (Tyr 397) were studied in the IBC cell lines FC-IBC02, KPL4, SUM190, and SUM149 treated with 1000 nM CEP-37440 during different time points. Control, cells grow in media without CEP-37440. Standard errors (SE) are indicated. FAK1 focal adhesion kinase 1, IBC inflammatory breast cancer

To test the effect of CEP-37440 on the autophosphorylation of FAK1 and its Tyr 397 site, IBC cell lines were treated with 1000 nM CEP-37440 during different time intervals (0 h, 48 h, 72 h, 96 h, and 120 h) and total FAK1 and phospho-FAK1 (Tyr 397) were studied (Fig. 4). In FC-IBC02 cells treated with CEP-37440, the levels of total FAK1 were similar compared to the control without treatment (0.5 ng to 0.6 ng total FAK1/μg protein) (Fig. 4a), but the levels of phospho-FAK1 decreased over time in the cells treated with CEP-37440, reaching a value lower than 0.002 units phospho-FAK1/μg protein at 72 to 120 h of treatment (Fig. 4b). In SUM190 cells, the levels of total FAK1 decreased to approximately 50 % in the cells treated with 1000 nM of CEP-37440 compared to the control without treatment (Fig. 4a), and the phospho-FAK1 (Tyr 397) decreased from 0.07 units/μg protein in the control to 0.0015 units/μg protein in the CEP-37440-treated cells after 48 to 120 h of treatment (Fig. 4b). In both FC-IBC02 and SUM190 cells, the levels of phospho-FAK1 (Tyr 397) reached similar levels (0.0015 to 0.002 units/μg protein) after the treatment with 1000 nM CEP-37440 for 48 h (Fig. 4b).

In KPL4 cells, the levels of total FAK1 were similar between cells treated with CEP-37440 and the control without treatment (Fig. 4a), however, there was a significant decrease in phospho-FAK1 (Tyr 397) after CEP-37440 treatment from 2.5 % in the control without treatment to 1.4 % in cells treated with CEP-37440 (Fig. 4b and 4c). In SUM149, the levels of total FAK1 in treated cells were similar to the control during the first 72 h of treatment, decreasing from 0.4–0.2 ng total FAK1/μg protein after 96–120 h of CEP-37440 treatment (Fig. 4a); however there was a slightly decrease of phospho-FAK1, decreasing from 0.019 Units phospho-FAK1/ μg protein in the control to 0.008-0.009 Units phospho-FAK1/ μg protein at 96-120 h of CEP-37440 treatment (Fig. 4b). In SUM149, there was a decrease from 4.9 % phospho-FAK1 in the control without treatment to 3.8 % phospho-FAK1 after 120 h of CEP-37440 treatment (Fig. 4c).

In conclusion, low concentration of CEP-37440 (1000 nM) was able to decreased phospho-FAK1 by half in FC-IBC02, SUM190, and KPL4 cells after 48 h of treatment compared to the controls without treatment, while in SUM149, it only decreased slightly.

### Expression arrays analyses of FC-IBC02 cells treated with CEP-37440

**Fig. 5:**
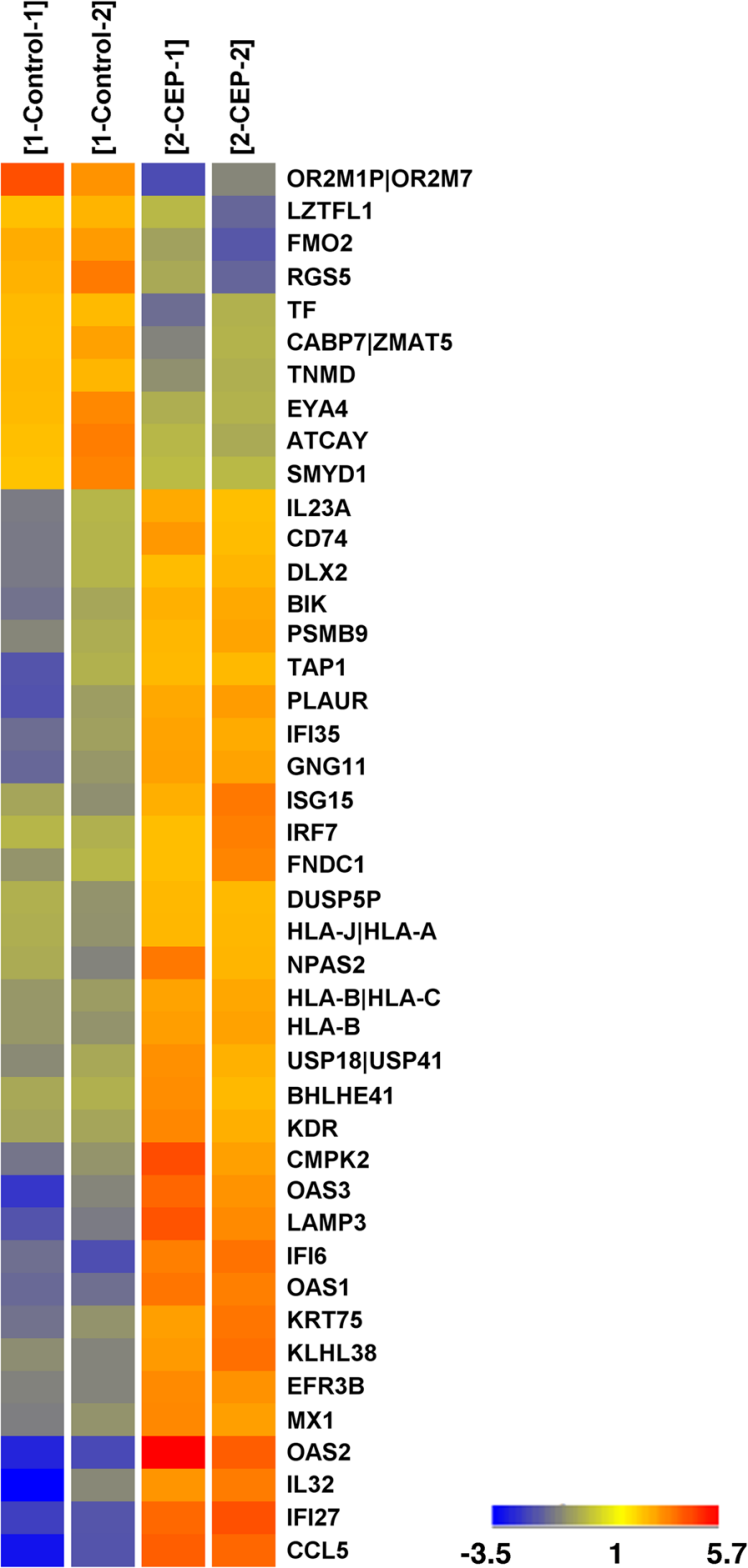
Heat map of FC-IBC02 cells treated with CEP-37440. FC-IBC02 cells were treated with 1000 nM CEP-37440 for 48 h, RNA was isolated and expression arrays were performed. RNA isolate from FC-IBC02 without treatment was used as control. *Red*, *yellow* and *blue colors* indicate levels above, at, and below mean expression, respectively. In the color scale, fold induction values are indicated

FC-IBC02 IBC cells were selected for expression array analyses because our previous results showed that CEP-37440 at low concentration (1000 nM) was able to completely inhibit the proliferation of these cells in vitro. Cells were treated with 1000 nM CEP-37440 for 48 h, RNA was isolated from the cells, and expression studies were performed. The microarray data have been deposited into the NCBI’s gene expression omnibus (GEO) data sets (GSE73285). A total of 43 genes showed changes in their expression when cells were treated with CEP-37440 compared to the control (2.0-fold, *p* = 0.05), with ten downregulated and 33 upregulated genes (Fig. 5 and Table 2). Using IPA, the major functions affected by CEP-37440 were cell death and survival, cellular growth and proliferation, and motility. Among key upregulated genes, those related to interferon signaling and cytokines were upregulated as follows: IFI27 (4.3-fold), IFI6 (3.5-fold), IFI35 (2.4-fold), IRF7 (2.1-fold), CCL5 (4.6-fold), IL32 (3.6-fold), and IL23A (2.1-fold). Similarly, the expression of interferon-stimulated genes was upregulated, as well: OAS2 (5.8-fold), OAS3 (3.5-fold), OAS1 (3.3-fold), MX1 (2.6-fold), and ISG15 (2.4-fold). CEP-37440 upregulated two other genes related to cell death and survival: BIK (2.3-fold) and KDR (2.3-fold).

**Table 2:** Genes dysregulated in FC-IBC02 cells by CEP-37440

Gene symbol	Gene description	Fold induction	*p* value
IFI27	Interferon, alpha-inducible protein 27	4.3	0.002
IFI6	Interferon, alpha-inducible protein 6	3.5	0.003
IFI35	Interferon-induced protein 35	2.4	0.013
IRF7	Interferon regulatory factor 7	2.1	0.036
CCL5	Chemokine ligand 5 (Rantes)	4.6	0.008
IL32	Interleukin 32	3.6	0.044
IL23A	Interleukin 23, alpha subunit P19	2.1	0.028
OAS2	2'-5'-oligoadenylate synthetase 2, 69/71 kDa	5.8	0.016
OAS3	2'-5'-oligoadenylate synthetase 3, 100 kDa	3.5	0.022
OAS1	2'-5'-oligoadenylate synthetase 1, 40/46 kDa	3.3	4.70E-04
MX1	MX dynamin-like GTPase 1	2.6	0.005
ISG15	ISG15 ubiquitin-like modifier	2.4	0.021
BIK	BCL2-interacting killer (apoptosis-inducing)	2.3	0.016
KDR	Kinase insert domain receptor	2.3	0.012
LAMP3	Lysosomal-associated membrane protein 3	3.6	0.013
CMPK2	Cytidine monophosphate kinase 2	3.0	0.03
KLHL38	Kelch-like family member 38	2.8	0.009
EFR3B	EFR3 homolog B	2.8	3.14E-04
KRT75	Keratin 75, type II	2.8	0.013
GNG11	Guanine nucleotide-binding protein, gamma 11	2.6	0.010
PLAUR	Plasminogen activator, urokinase receptor	2.6	0.024
HLA-B	Major histocompatibility complex, class I, B	2.4	2.50E-04
NPAS2	Neuronal PAS domain protein 2	2.4	0.031
HLA-B|HLA-C	Major histocompatibility complex,	2.3	2.38E-04
TAP1	Transporter 1, ATP-binding cassette, sub-family B	2.3	0.048
USP18|USP41	Ubiquitin specific peptidase 18/41	2.3	0.013
FNDC1	Fibronectin type III domain-containing 1	2.2	0.033
CD74	CD74 molecule	2.2	0.03
PSMB9	Proteasome subunit, beta type, 9	2.2	0.013
BHLHE41	Basic helix-loop-helix family, member E41	2.1	0.018
DLX2	Distal-less homeobox 2	2.1	0.025
DUSP5P	Dual specificity phosphatase 5 pseudogene 1	2.0	0.007
HLA-J|HLA-A	Major histocompatibility complex	2.0	0.007
OR2M1P|OR2M7	Olfactory receptors	−3.5	0.023
FMO2	Flavin containing monooxygenase 2	−2.6	0.026
RGS5	Regulator of G protein signaling 5	−2.6	0.036
TF	Transferrin	−2.2	0.031
CABP7	Calcium-binding protein 7	−2.2	0.021
LZTFL1	Leucine zipper transcription factor-like 1	−2.2	0.044
ATCAY	Ataxia, cerebellar, Cayman type	−2.1	0.038
EYA4	EYA transcriptional coactivator and phosphatase 4	−2.1	0.023
SMYD1	SET and MYND domain-containing 1	−2.0	0.041
TNMD	Tenomodulin	−2.0	0.007

Cells were treated with 1000 nM CEP-37440 during 48 h and expression studies were performed

### CEP-37440 inhibited breast tumor growth in the IBC breast tumor xenograft models

**Fig. 6:**
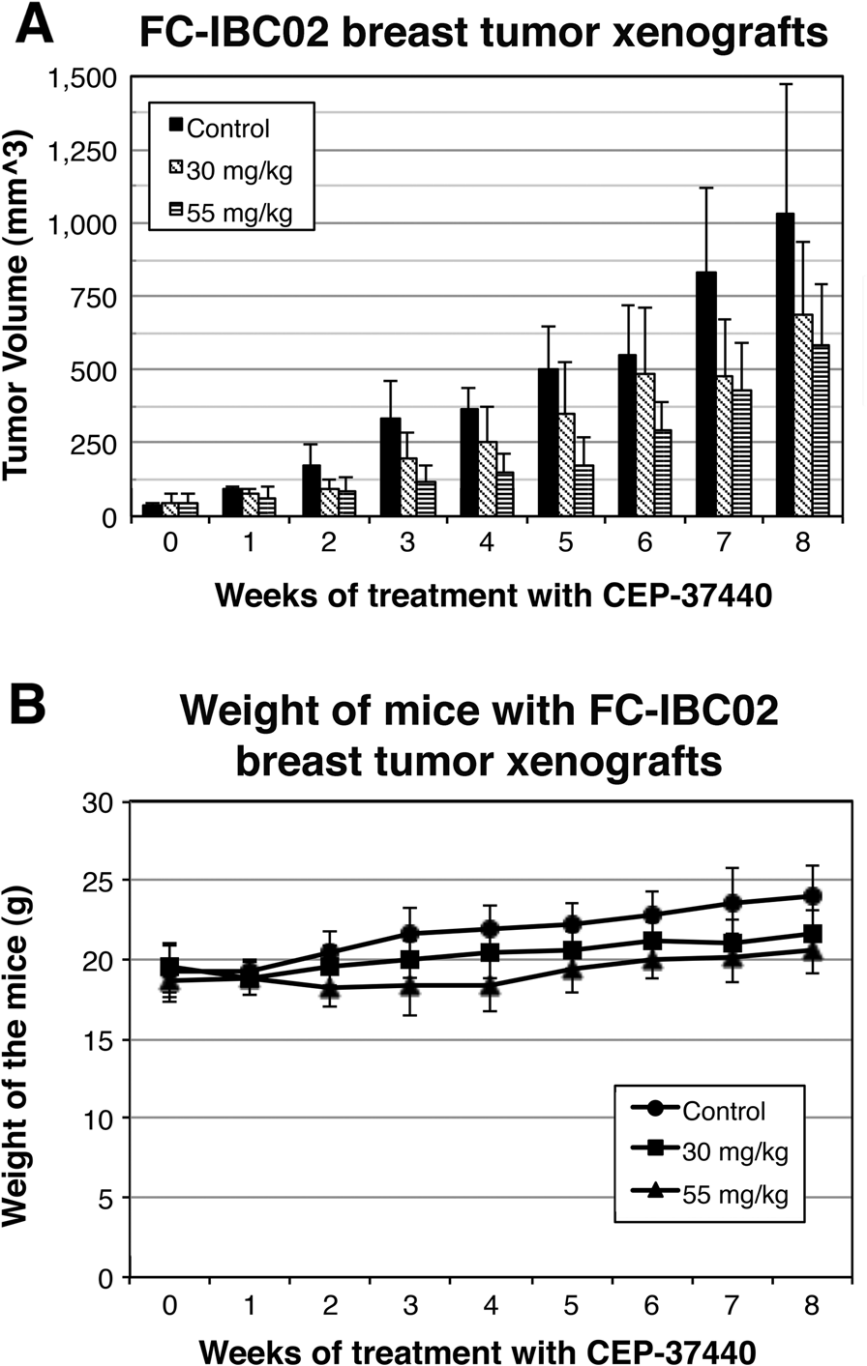
In vivo studies: effect of CEP-37440 on mice with FC-IBC02 breast tumor xenografts. Mice were injected with 10^6^ FC-IBC02 cells in the mammary fat pad; when the breast tumor xenografts reached approximately 50 mm^3^, treatments with CEP-37440 began. Treatments with CEP-37440 were performed twice a day during 8 weeks. **a** Volumes of breast tumor xenografts in the control group (without treatment) or mice treated with 30 mg/kg or 55 mg/kg CEP-37440; **b** weights of the mice in the different groups. Standard deviations (StDEV) are indicated

**Fig. 7:**
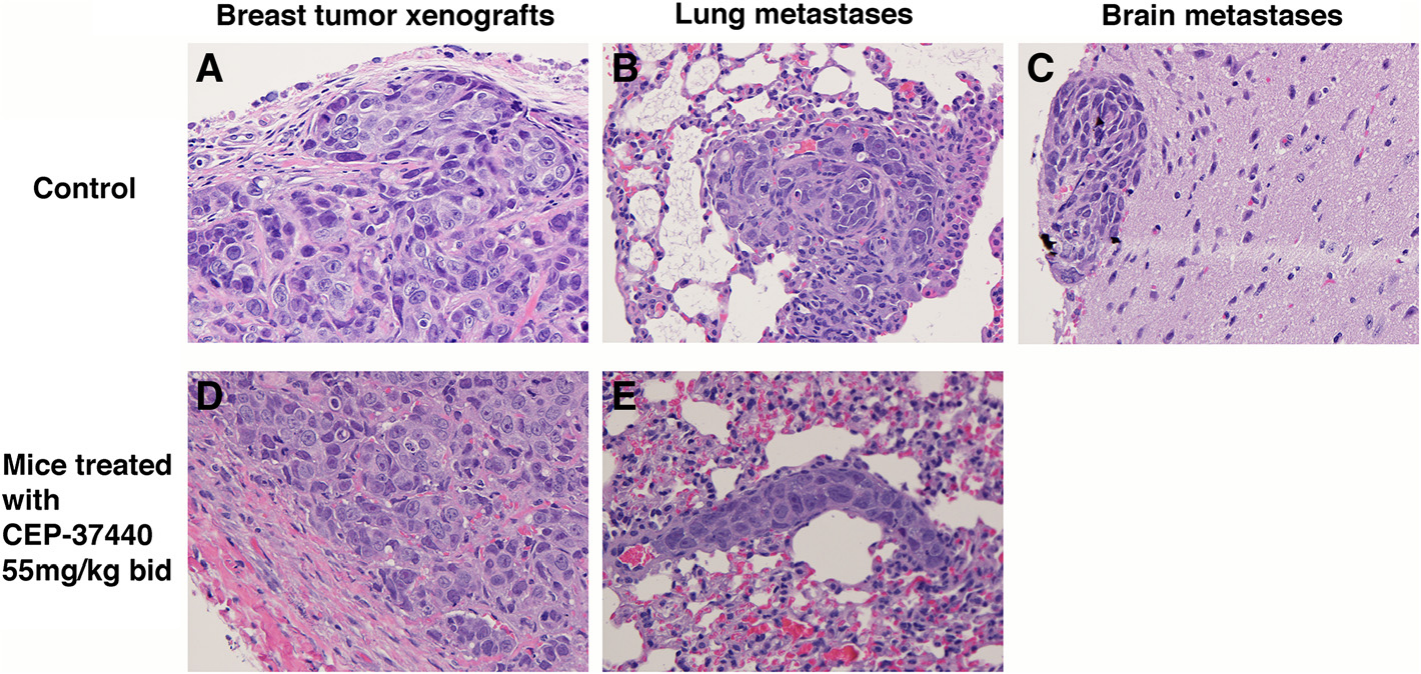
Histological specimens in mice harboring FC-IBC02 breast tumor xenograft. Specimens from the control group (without treatment): **a** breast tumor xenograft, **b** lung metastases, and **c **brain metastasis. Specimens from mice treated with 55 mg/kg CEP-37440: **d** breast tumor xenograft, and **e** lung metastases. Multiple metastatic sites were observed in the lungs of the control and treated mice. A single focus of metastatic tumor cells in the brain was found in two mice from the control group; no brain metastases were found in the mice treated with CEP-37440. Hematoxilin and eosin (H&E) stain. Magnification: ×40

CEP-37440 was tested in vivo using the triple-negative FC-IBC02 and SUM149 and the ErbB2-positive SUM190 IBC breast xenograft models. The IBC cells were injected in the mammary fat pad of female SCID mice, and treatment with CEP-37440 began once the breast tumor xenografts reached approximately 50–100 mm^3^. All of the mice injected with FC-IBC02 or SUM149 developed breast tumor xenografts, and two doses of CEP-37440 (30 mg/kg and 55 mg/kg) were tested in these mice. In the animals harboring FC-IBC02 breast tumor xenografts, mice treated with 55 mg/kg bid CEP-37440 showed smaller tumor breast xenografts compared to the control group that did not receive the drug (Fig. 6). The breast tumor growth was significantly lower in the group treated with 55 mg/kg bid CEP-37440 than the control group without treatment over the 7-week study (*p* ≤ 0.001) (Additional file 7: Figure S4 and Additional file 8: Table S4). Also, mice treated with 55 mg/kg bid showed smaller tumors than the mice treated with 30 mg/kg bid CEP-37440 over the entire duration of the study (Additional file 7: Figure S4). At the end of the study, there was approximately 33 % reduction in the FC-IBC02 breast tumor xenograft size after the mice were treated with 55 mg/kg bid CEP-37440 (week 8; 40 days of treatment) (Fig. 6a) (*p* = 0.128). Furthermore, there was approximately 21 % reduction in the FC-IBC02 breast tumor xenografts size after the mice were treated with 30 mg/kg bid CEP-37440 at week 8 (40 days of treatment) (*p* = 0.380) (Fig. 6a). FC-IBC02 mice treated with CEP-37440 showed no signs of toxicity, such as hair loss or significant weight loss compared to the control group without treatment (Fig. 6b). Importantly, although our previous experiments showed that 20 % of the mice with FC-IBC02 breast xenografts developed spontaneous metastatic brain tumors (Fig. 7c), none of the mice treated with CEP-37440 (30 mg/kg or 55 mg/kg) developed brain tumors. No differences in the number of metastatic sites in the lungs were found when comparing the 55 mg/kg CEP-37440-treated mice (Fig. 7e) and the control group (Fig. 7b) .

**Fig. 8:**
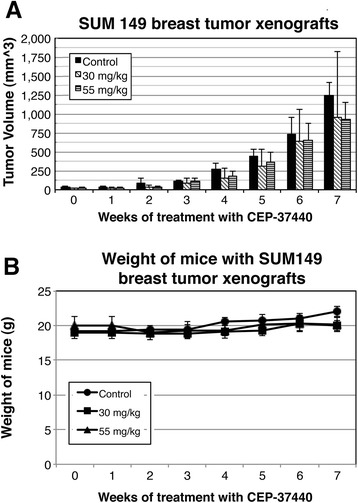
In vivo studies: effect of CEP-37440 on mice with SUM149 breast tumor xenografts. Mice were injected with 10^6^ SUM149 cells in the mammary fat pad; when the breast tumor xenografts reached approximately 50 mm^3^, treatments with CEP-37440 began. Treatments with CEP-37440 were performed twice a day during 7 weeks (35 days of treatment). **a** Volumes of breast tumor xenografts in the control group (without treatment) or mice treated with 30 mg/kg or 55 mg/kg CEP-37440; **b** weights of the mice in the different groups. Standard deviations (StDEV) are indicated

In animals harboring SUM149 breast tumor xenografts, mice treated with CEP-37440 showed smaller tumor breast xenografts compared to the control group that did not receive the drug (Fig. 8a). The breast tumor growth was significantly lower in the mice treated with CEP-37440 than in the control group without treatment over the 7-week study, although the growth of the tumors was lower in the mice treated with 30 mg/kg bid than with 55 mg/kg bid CEP-37440 (Additional file 9: Figure S5 and Additional file 10: Table S5). At the end of the study (week 7, 35 days of treatment), there was approximately 43 % reduction in the SUM149 breast tumor xenograft size after the mice were treated with 30 mg/kg bid CEP-37440 (*p* = 0.021) and approximately 23 % reduction in the SUM149 breast tumor xenografts size after the mice were treated with 55 mg/kg bid CEP-37440 at week 7 (*p* = 0.302). SUM149 breast tumor xenograft mice treated with CEP-37440 showed no significant weight loss compared to the control group that was not treated with CEP-37440 (Fig. 8b).

**Fig. 9:**
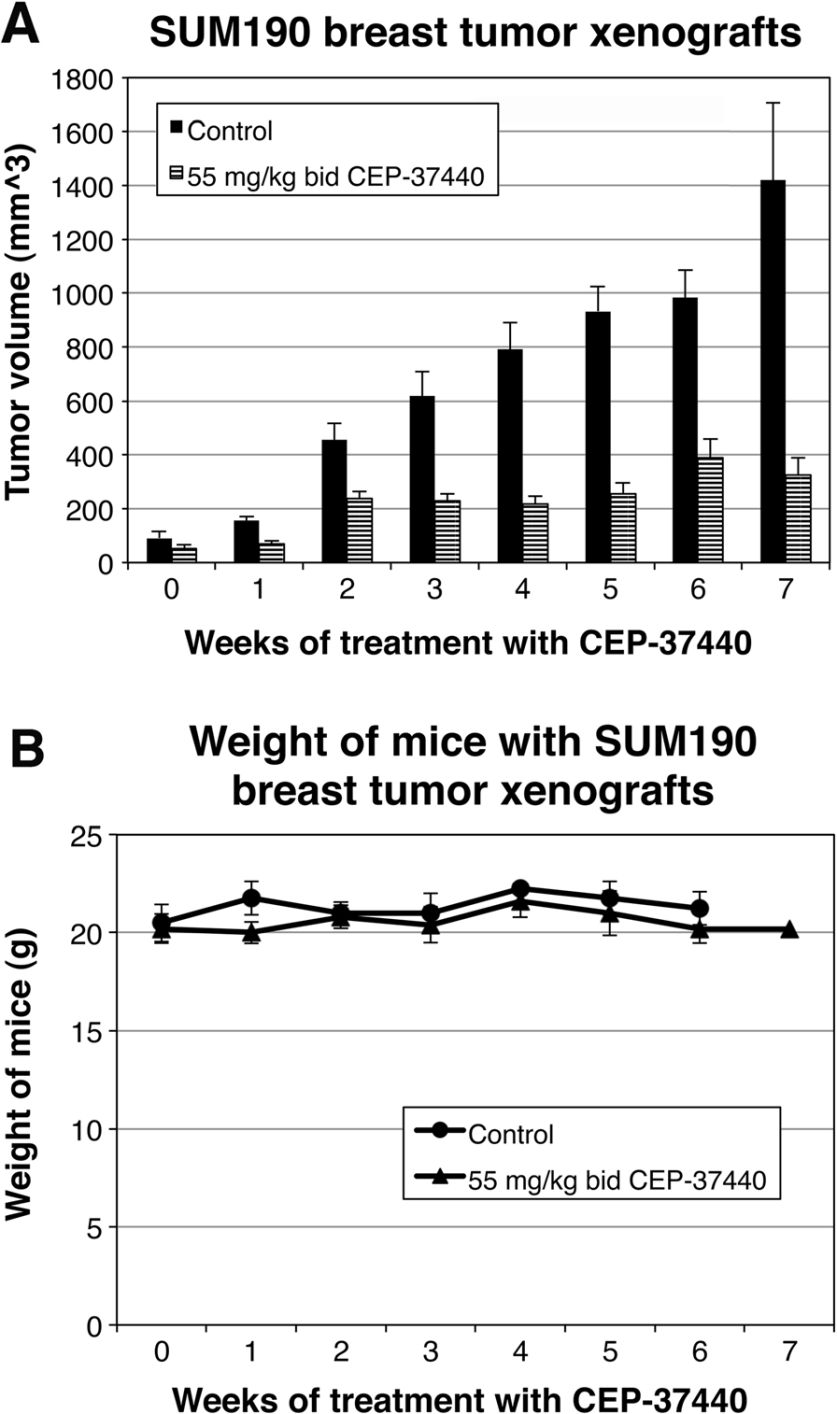
In vivo studies: effect of CEP-37440 on mice with SUM190 breast tumor xenografts. Mice were injected with 10^6^ SUM190 cells in the mammary fat pad; when the breast tumor xenografts reached approximately 100 mm^3^, treatment with 55 mg/kg bid CEP-37440 began. Treatments with CEP-37440 were performed twice a day during 7 weeks (35 days of treatment). **a** Volumes of breast tumor xenografts in the control group (without treatment) or mice treated with 55 mg/kg CEP-37440; **b** weights of the mice in the different groups. Standard errors (SE) are indicated

CEP-37440 was also tested in mice harboring SUM190 ErbB2-positive breast tumor xenografts. A significant difference in the size of the SUM190 breast tumor xenografts was observed in the treated group when compared to mice in the control group that did not receive the drug (Fig. 9a). There was 79.7 % reduction in the SUM190 breast tumor xenograft size after the mice were treated with 55 mg/kg bid CEP-37440 for 35 days (week 7) (*p* = 0.001) (Additional file 11: Table S6). There were no significant differences between the weight of the control and CEP-37440-treated mice (Fig. 9b).

## Discussion

Our studies showed that low concentrations of CEP-37440 specifically decreased the proliferation of the IBC cell lines FC-IBC02, SUM190, and KPL4 without affecting proliferation of normal breast epithelial cells. These IBC cells were sensitive to low concentrations of CEP-37440 and their sensitivity was independent of the cell subtype (triple-negative or ErbB2-positive). At higher concentrations, CEP-37440 also inhibited the proliferation of the IBC cell line MDA-IBC03 and the triple-negative non-IBC cell lines MDA-MB-231 and MDA-MB-468. Since CEP-37440 is a dual inhibitor of FAK1 and ALK, we studied the expression of both proteins in the IBC cell lines and found that they expressed FAK1 but not ALK. We demonstrated that CEP-37440 decreased the cell proliferation of FC-IBC02, SUM190, and KPL4 by blocking the autophosphorylation kinase activity of FAK1 (Tyr 397).

We previously showed that IBC cells exhibit amplification of the chromosomal arm 8q where FAK1 is located (8q24.3) [10]. However, gene expression studies using arrays did not show any correlation between copy number and RNA expression in these IBC cell lines [10]. Furthermore, our present work did not find a correlation between elevated total FAK1 expression at the protein level and FAK1 gene amplification in either IBC cells or triple-negative non-IBC cell lines. Increased dosage of the FAK1 gene is invariantly observed in the cell lines derived from human cancers of lung, breast, colon, and invasive squamous cell carcinomas [22]. However, elevated FAK1 protein expression is not always correlated with amplification of the FAK1 gene. In human head and neck squamous cell carcinoma, not all cases with an amplification of the FAK1 gene display FAK1 protein overexpression, implicating a sophisticated posttranscriptional regulation involved in FAK1 expression and function [23].

In vivo studies showed that CEP-37440 significantly decreased breast tumor growth in the SUM190 and FC-IBC02 mouse xenograft models, and the tumor growth inhibition was dose and time dependent. Mice harboring SUM190 and FC-IBC02 breast tumor xenografts showed 79.7 % and 33 % TGI, respectively, when treated with 55 mg/kg bid CEP-37440 for 35–40 days. The tumor breast xenografts of mice treated with CEP-37440 showed lower levels of phospho-FAK1 (Tyr 397) than the breast tumor xenografts in the control groups (data not shown). Although the IBC cell line SUM149 showed in vitro only a slight response to high concentrations of the drug, CEP-37440 reduced the size of the primary tumor in the mice harboring SUM149 breast tumor xenografts, and results were better with 30 mg/kg bid than 55 mg/kg bid CEP-37440. Since there was no total regression of the primary tumor or metastatic sites in the lungs in mice harboring IBC xenografts, our results suggest that a combination therapy approach would be more effective for IBC patients than CEP-37440 alone. Preclinical studies showed that the combination therapy approach using inhibitors of FAK1 with other signaling pathways increased the efficacy of single inhibitors [22, 23]. These types of combinatorial studies have not been conducted with CEP-37440 in preclinical models of IBC or triple-negative breast cancer (TNBC).

None of the FC-IBC02 breast xenograft mice treated with CEP-37440 developed brain metastases in contrast to 20 % in the control group. This data suggests that CEP-37440 is able to cross the blood-brain barrier. Related work by another group found that TAE226, another small molecule inhibitor targeting the ATP-binding site of FAK1, increased apoptosis of glioblastoma, an infiltrative brain tumor, and inhibited tumor growth [24].

The overexpression and phosphorylation of FAK1 on Tyr 397 is frequently associated with tumor metastasis as well as poor patient prognosis [25–28], indicating a critical role for activated FAK1 in tumor progression and malignancy. Tyrosine 397 is the main autophosphorylation site of FAK1, leading to activation of its intrinsic kinase function as well as its downstream signaling players, and providing a high-affinity binding site for the SH2 domain of Src family kinases [29, 30]. Furthermore, phospho-FAK1 binds to the p85 subunit of phosphatidylinositol 3-kinase (PI3K) at the Tyr 397 autophosphorylation site. Phospholipid production stimulated by phospho-FAK1 and activation of PIK3 can stimulate AKT kinase, which inhibits apoptosis by regulating various cell death cascade proteins [31]. In addition to its roles as a cytoplasmic kinase, recent studies revealed that FAK1 can translocate to the nucleus where it can influence the expression of chemokines, which are secreted to the surrounding environment [32]. These proteins recruit regulatory T cells (Treg) into tumors, which in turn suppress the CD8 antitumor response. Therefore, nuclear FAK1 signaling in cancer cells can help establish an environment within the tumor that supports survival and growth. FAK1 kinase inhibitors target mechanisms of immune suppression and may therefore represent a form of effective “immune-modulatory” therapy that reduces regulatory T cells in the tumor environment [32]. We showed that CEP-37440 treatment of the IBC cell line FC-IBC02 increased the expression of multiple genes related to interferon signaling and cytokines.

In a recent study, FAK1 expression was analyzed in breast primary tumor samples from stages III–IV patients and a significant association between high FAK1 expression in the primary tumor, lymphovascular invasion, and triple-negative phenotype was found. In addition, a strong positive correlation was observed between high FAK1 expression and shorter overall survival and progression-free survival in patients with metastatic tumors [33]; however, it was not specified by the authors if IBC samples were included in the study. The role of ALK in IBC is controversial, with some studies showing amplification of ALK in IBC and in others showing no amplification [34, 35], and the present work showed that none of the IBC cell lines expressed the ALK protein.

FAK1 is one of the most attractive tyrosine kinase targets in cancer therapy since it plays a role in signal transduction and in the development of numerous human tumors, including breast, colon, thyroid, prostate, pancreas, and brain cancers [36]. Besides CEP-37440, several other small molecule inhibitors of FAK1 that target the ATP-binding site and block FAK1 kinase activity have been developed. One of these inhibitors, PF-562,271 from Pfizer decreased tumor growth in multiple xenograft models [37, 38], and was shown to be effective in phase I trials [39]; however, it showed nonlinear pharmacokinetics and was henceforth discontinued. VS-6063 (defactinib) from Verastem showed more favorable pharmacokinetics, and in a phase I trial it demonstrated a favorable response in some patients with ovarian, colorectal, or bile duct cancer. Other FAK1 inhibitors, including VS-4718 (or PND-1186) [40], VS-5095, and GSK2256098, are in early clinical trials.

Finally, our in vivo experiments demonstrated that animals treated with CEP-37440 did not experience reduced body weight or hair loss, suggesting that CEP-37440 was not toxic for mice at a dose of 30 or 55 mg/kg/bid. CEP-37440 is currently in a phase I dose-escalation clinical trial in patients with solid tumors (www.clinicaltrials.gov NCT01922752). Based upon the preclinical studies described above and the data available for other FAK1 inhibitors, CEP-37440 may have clinical applications as part of a combinatorial therapy against IBC.

## Conclusions

These results suggest that CEP-37440 could be effective against IBC cells that express phospho-FAK1 (Tyr 397) if the drug is able to decrease the level of phospho-FAK (Tyr-397). Our results also suggest that combinational therapies could be more effective than using CEP-37440 as a single agent.

## Additional files

Additional file 1: Figure S1. FC-IBC02 cell proliferation assays: estimated time trends in response to different CEP-37440 concentrations in the triple-negative IBC cell line FC-IBC02. (DOC 54 kb)

Additional file 2: Table S1. FC-IBC02 cell proliferation assays: comparisons from the LME model and time trend estimates by CEP-37440 doses. (DOC 81 kb)

Additional file 3: Figure S2. KPL4 cell proliferation assays: estimated time trends in response to CEP-37440 concentrations in the ErbB2-positive IBC cell line KPL4. (DOC 76 kb)

Additional file 4: Table S2. KPL4 cell proliferation assays: comparisons from the LME model for log-transformed responses and time trend estimates. (DOC 56 kb)

Additional file 5: Figure S3. SUM190 cell proliferation assays: estimated time trends in response to CEP-37440 concentration in the ErbB2-positive IBC cell line SUM190. (DOC 55 kb)

Additional file 6: Table S3. SUM190 cell proliferation assays: comparisons from the LME model for log-transformed responses and time trend estimates. (DOC 83 kb)

Additional file 7: Figure S4. In vivo studies using FC-IBC02 xenograft model: log-transformed tumor volumes and estimated time trends in each group from the LME model. (DOC 44 kb)

Additional file 8: Table S4. In vivo studies using FC-IBC02 xenograft model: results from the LME model and CEP-37440 treatment comparisons. (DOC 50 kb)

Additional file 9: Figure S5. In vivo studies using SUM149 xenograft model: log-transformed tumor volumes and estimated time trends in each group from the LME model. (DOC 393 kb)

Additional file 10: Table S5. In vivo studies using SUM149 xenograft model: results from the LME model and CEP-37440 treatment comparisons. (DOC 56 kb)

Additional file 11: Table S6. In vivo studies using SUM190 xenograft models. (DOC 39 kb)

Additional file 12: Supplementary Materials and Methods. Detail description of materials and methods. (DOCX 17 kb)

## Abbreviations

ALK: anaplastic lymphoma kinase
bid: twice a day
DMSO: dimethyl sulfoxide
EGFR: epidermal growth factor receptor
ELISA: enzyme-linked immunosorbent assay
ErbB2 or HER2: human epidermal growth factor receptor 2
ESR: estrogen receptor
FAK1 (PTK2 or FAK): focal adhesion kinase
GI_50_: drug concentration required to reduce growth rates to 50 %
H&E: hematoxylin and eosin
IBC: inflammatory breast cancer
IPA: Ingenuity Pathway Analysis
LME: linear mixed effects
MTS: 3-(4,5-dimethylthiazol-2-yl)-5-(3-carboxymethoxyphenyl)-2-(4-sulfophenyl)-2H-tetrazolium salt
NPM: nucleophosmin
PBS: phosphate-buffered saline
PgR: progesterone receptor
PI3K: phosphatidylinositol 3-kinase
PMSF: phenylmethanesulfonyl fluoride
SCID: severe combined immune-deficient
SE: standard error of the mean
StDEV: standard deviation
TGI: tumor growth inhibition
TNBC: triple-negative breast cancer
Treg: T regulatory cells
VEGFR: vascular epidermal growth factor receptor
