# Supplementary material for: The effects of CEP-37440, an inhibitor of focal adhesion kinase, in vitro and in vivo on inflammatory breast cancer cells
Source: Breast Cancer Res. 2016 Mar 24;18:37. doi: 10.1186/s13058-016-0694-4 (PMC4806466; doi:10.1186/s13058-016-0694-4)
Supplement: Supplementary file 1 — FC-IBC02 cell proliferation assays: estimated time trends in response to different CEP-37440 concentrations in the triple-negative IBC cell line FC-IBC02. (DOC 54 kb) [file 13058_2016_694_MOESM1_ESM.doc]

**
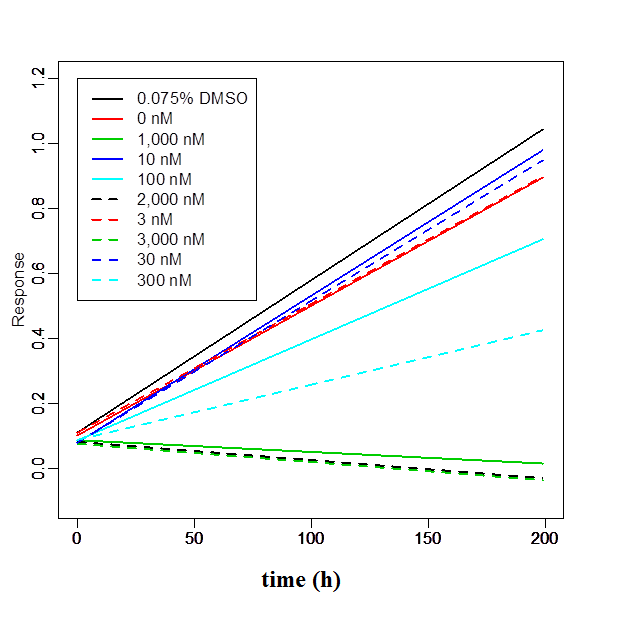
**

**Additional file 1: Figure S1.** FC-IBC02 cell proliferation assays: Estimated time trends in response to different CEP-37440 concentrations in the triple negative IBC cell line FC-IBC02.
