# Supplementary material for: The effects of CEP-37440, an inhibitor of focal adhesion kinase, in vitro and in vivo on inflammatory breast cancer cells
Source: Breast Cancer Res. 2016 Mar 24;18:37. doi: 10.1186/s13058-016-0694-4 (PMC4806466; doi:10.1186/s13058-016-0694-4)
Supplement: Supplementary file 2 — FC-IBC02 cell proliferation assays: comparisons from the LME model and time trend estimates by CEP-37440 doses. (DOC 81 kb) [file 13058_2016_694_MOESM2_ESM.doc]

| **Comparison** | **Difference** | **LL 95% CI** | **UL 95% CI** | **p-value** |
| --- | --- | --- | --- | --- |
| Intercept: Dose 0 nM vs. 0.075% DMSO | -0.009 | -0.118 | 0.100 | 0.871 |
| Intercept: Dose 1,000 nM vs. 0.075% DMSO | -0.024 | -0.128 | 0.081 | 0.653 |
| Intercept: Dose 10 nM vs. 0.075% DMSO | -0.031 | -0.141 | 0.079 | 0.574 |
| Intercept: Dose 100 nM vs. 0.075% DMSO | -0.024 | -0.133 | 0.084 | 0.654 |
| Intercept: Dose 2,000 nM vs. 0.075% DMSO | -0.029 | -0.133 | 0.075 | 0.584 |
| Intercept: Dose 3 nM vs. 0.075% DMSO | 0.000 | -0.110 | 0.109 | 0.994 |
| Intercept: Dose 3,000 nM vs. 0.075% DMSO | -0.035 | -0.139 | 0.070 | 0.510 |
| Intercept: Dose 30 nM vs. 0.075% DMSO | -0.031 | -0.141 | 0.078 | 0.572 |
| Intercept: Dose 300 nM vs. 0.075% DMSO | -0.022 | -0.129 | 0.084 | 0.677 |
| Slope: Dose 0 nM vs. 0.075% DMSO | -0.0007 | -0.0018 | 0.0004 | 0.221 |
| Slope: Dose 1,000 nM vs. 0.075% DMSO | -0.0050 | -0.0061 | -0.0040 | <0.001 |
| Slope: Dose 10 nM vs. 0.075% DMSO | -0.0002 | -0.0013 | 0.0010 | 0.775 |
| Slope: Dose 100 nM vs. 0.075% DMSO | -0.0016 | -0.0027 | -0.0005 | 0.006 |
| Slope: Dose 2,000 nM vs. 0.075% DMSO | -0.0052 | -0.0063 | -0.0042 | <0.001 |
| Slope: Dose 3 nM vs. 0.075% DMSO | -0.0007 | -0.0019 | 0.0004 | 0.208 |
| Slope: Dose 3,000 nM vs. 0.075% DMSO | -0.0052 | -0.0063 | -0.0042 | <0.001 |
| Slope: Dose 30 nM vs. 0.075% DMSO | -0.0003 | -0.0015 | 0.0008 | 0.579 |
| Slope: Dose 300 nM vs. 0.075% DMSO | -0.0030 | -0.0041 | -0.0019 | <0.001 |
| **Time trends** | **Estimate** | **LL 95% CI** | **UL 95% CI** | **p-value** |
| Intercept: Dose 0.075% DMSO | 0.111 | 0.033 | 0.189 | 0.006 |
| Intercept: Dose 0 nM | 0.102 | 0.026 | 0.178 | 0.009 |
| Intercept: Dose 1,000 nM | 0.087 | 0.019 | 0.156 | 0.014 |
| Intercept: Dose 10 nM | 0.080 | 0.003 | 0.157 | 0.041 |
| Intercept: Dose 100 nM | 0.087 | 0.012 | 0.162 | 0.024 |
| Intercept: Dose 2,000 nM | 0.082 | 0.014 | 0.151 | 0.020 |
| Intercept: Dose 3 nM | 0.111 | 0.034 | 0.187 | 0.005 |
| Intercept: Dose 3,000 nM | 0.077 | 0.008 | 0.145 | 0.030 |
| Intercept: Dose 30 nM | 0.080 | 0.003 | 0.157 | 0.042 |
| Intercept: Dose 300 nM | 0.089 | 0.016 | 0.161 | 0.017 |
| Slope: Dose 0.075% DMSO: Time | 0.0047 | 0.0039 | 0.0055 | <0.001 |
| Slope: Dose 0 nM: Time | 0.0040 | 0.0032 | 0.0048 | <0.001 |
| Slope: Dose 1,000 nM: Time | -0.0004 | -0.0010 | 0.0002 | 0.233 |
| Slope: Dose 10 nM: Time | 0.0045 | 0.0037 | 0.0053 | <0.001 |
| Slope: Dose 100 nM: Time | 0.0031 | 0.0024 | 0.0039 | <0.001 |
| Slope: Dose 2,000 nM: Time | -0.0006 | -0.0012 | 0.0000 | 0.065 |
| Slope: Dose 3 nM: Time | 0.0040 | 0.0032 | 0.0047 | <0.001 |
| Slope: Dose 3,000 nM: Time | -0.0006 | -0.0012 | 0.0000 | 0.064 |
| Slope: Dose 30 nM: Time | 0.0044 | 0.0036 | 0.0052 | <0.001 |
| Slope: Dose 300 nM: Time | 0.0017 | 0.0010 | 0.0024 | <0.001 |

**Additional file 2: Table S1.** FC-IBC02 cell proliferation assays: Comparisons from the LME model and time trend estimates by CEP-37440 doses.
