# Supplementary material for: The effects of CEP-37440, an inhibitor of focal adhesion kinase, in vitro and in vivo on inflammatory breast cancer cells
Source: Breast Cancer Res. 2016 Mar 24;18:37. doi: 10.1186/s13058-016-0694-4 (PMC4806466; doi:10.1186/s13058-016-0694-4)
Supplement: Supplementary file 3 — KPL4 cell proliferation assays: estimated time trends in response to CEP-37440 concentrations in the ErbB2-positive IBC cell line KPL4. (DOC 76 kb) [file 13058_2016_694_MOESM3_ESM.doc]

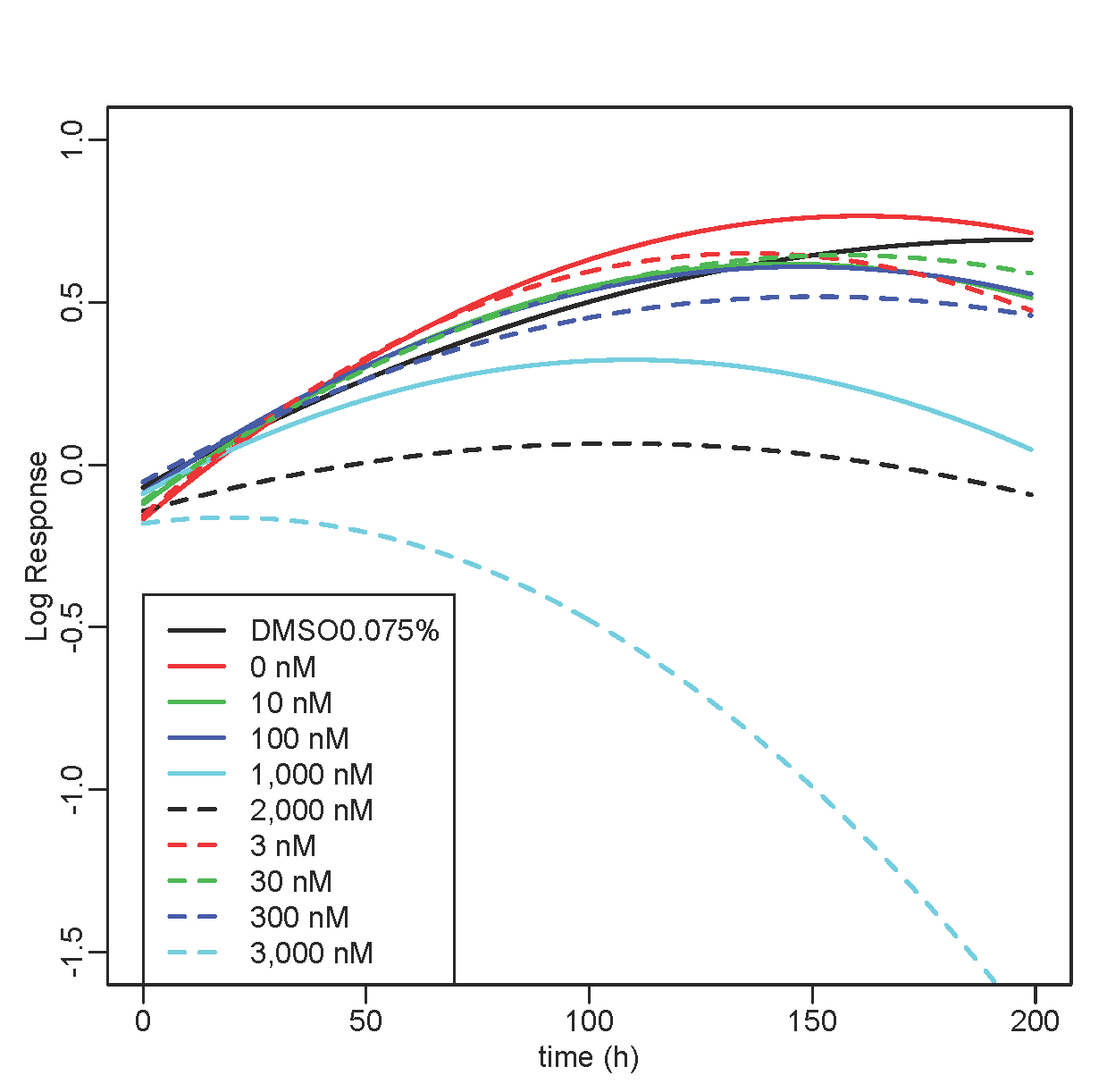


**Additional file 3: Figure S2.** KPL4 cell proliferation assays: Estimated time trends in response to CEP-37440 concentrations in the ErbB2-positive IBC cell line KPL4.
