# Supplementary material for: The effects of CEP-37440, an inhibitor of focal adhesion kinase, in vitro and in vivo on inflammatory breast cancer cells
Source: Breast Cancer Res. 2016 Mar 24;18:37. doi: 10.1186/s13058-016-0694-4 (PMC4806466; doi:10.1186/s13058-016-0694-4)
Supplement: Supplementary file 4 — KPL4 cell proliferation assays: comparisons from the LME model for log-transformed responses and time trend estimates. (DOC 56 kb) [file 13058_2016_694_MOESM4_ESM.doc]

| **Time trends** | **Estimate** | **LL 95% CI** | **UL 95% CI** | **p-value** |
| --- | --- | --- | --- | --- |
| Intercept: Dose 0.075% DMSO | -0.070 | -0.366 | 0.227 | 0.641 |
| Intercept: Dose 0 nM | -0.168 | -0.471 | 0.135 | 0.273 |
| Intercept: Dose 10 nM | -0.121 | -0.425 | 0.182 | 0.428 |
| Intercept: Dose 100 nM | -0.088 | -0.392 | 0.215 | 0.564 |
| Intercept: Dose 1,000 nM | -0.089 | -0.393 | 0.214 | 0.558 |
| Intercept: Dose 2,000 nM | -0.143 | -0.446 | 0.160 | 0.350 |
| Intercept: Dose 3 nM | -0.156 | -0.460 | 0.147 | 0.307 |
| Intercept: Dose 30 nM | -0.112 | -0.416 | 0.191 | 0.461 |
| Intercept: Dose 300 nM | -0.053 | -0.356 | 0.251 | 0.730 |
| Intercept: Dose 3,000 nM | -0.181 | -0.484 | 0.123 | 0.238 |
| Slope: Dose 0.075% DMSO | 0.008 | 0.000 | 0.015 | 0.041 |
| Slope: Dose 0 nM | 0.012 | 0.004 | 0.019 | 0.003 |
| Slope: Dose 10 nM | 0.010 | 0.003 | 0.018 | 0.007 |
| Slope: Dose 100 nM | 0.009 | 0.002 | 0.017 | 0.013 |
| Slope: Dose 1,000 nM | 0.008 | 0.000 | 0.015 | 0.045 |
| Slope: Dose 2,000 nM | 0.004 | -0.003 | 0.011 | 0.292 |
| Slope: Dose 3 nM | 0.012 | 0.005 | 0.019 | 0.002 |
| Slope: Dose 30 nM | 0.010 | 0.002 | 0.017 | 0.011 |
| Slope: Dose 300 nM | 0.008 | 0.000 | 0.015 | 0.045 |
| Slope: Dose 3,000 nM | 0.002 | -0.005 | 0.009 | 0.610 |
| Quadr.coef: Dose 0.075% DMSO | -0.000019 | -0.000056 | 0.000018 | 0.305 |
| Quadr.coef: Dose 0 nM | -0.000036 | -0.000073 | 0.000001 | 0.056 |
| Quadr.coef: Dose 10 nM | -0.000035 | -0.000072 | 0.000002 | 0.061 |
| Quadr.coef: Dose 100 nM | -0.000032 | -0.000069 | 0.000005 | 0.089 |
| Quadr.coef: Dose 1,000 nM | -0.000034 | -0.000071 | 0.000003 | 0.068 |
| Quadr.coef: Dose 2,000 nM | -0.000018 | -0.000055 | 0.000019 | 0.323 |
| Quadr.coef: Dose 3 nM | -0.000044 | -0.000081 | -0.000007 | 0.021 |
| Quadr.coef: Dose 30 nM | -0.000031 | -0.000068 | 0.000006 | 0.100 |
| Quadr.coef: Dose 300 nM | -0.000025 | -0.000062 | 0.000012 | 0.180 |
| Quadr.coef: Dose 3,000 nM | -0.000049 | -0.000086 | -0.000012 | 0.011 |

**Additional file 4: Table S2.** KPL4 cell proliferation assays: Comparisons from the LME model for log-transformed responses and time trend estimates by CEP-37440 concentrations.
