# Supplementary material for: The effects of CEP-37440, an inhibitor of focal adhesion kinase, in vitro and in vivo on inflammatory breast cancer cells
Source: Breast Cancer Res. 2016 Mar 24;18:37. doi: 10.1186/s13058-016-0694-4 (PMC4806466; doi:10.1186/s13058-016-0694-4)
Supplement: Supplementary file 5 — SUM190 cell proliferation assays: estimated time trends in response to CEP-37440 concentration in the ErbB2-positive IBC cell line SUM190. (DOC 55 kb) [file 13058_2016_694_MOESM5_ESM.doc]

**
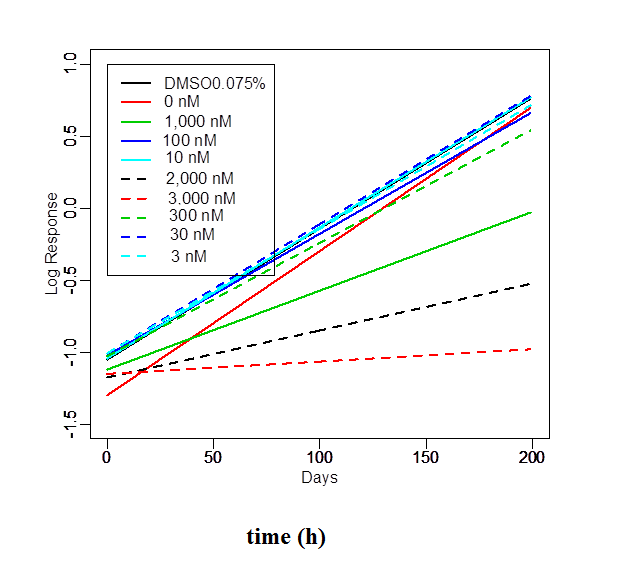
**

**Additional file 5: Figure S3.** SUM190 cell proliferation assays: Estimated time trends in response to CEP-37440 concentration in the ErbB2-positive IBC cell line SUM190.
