# Supplementary material for: The effects of CEP-37440, an inhibitor of focal adhesion kinase, in vitro and in vivo on inflammatory breast cancer cells
Source: Breast Cancer Res. 2016 Mar 24;18:37. doi: 10.1186/s13058-016-0694-4 (PMC4806466; doi:10.1186/s13058-016-0694-4)
Supplement: Supplementary file 6 — SUM190 cell proliferation assays: comparisons from the LME model for log-transformed responses and time trend estimates. (DOC 83 kb) [file 13058_2016_694_MOESM6_ESM.doc]

| **Comparison** | **Mean Diff.** | **LL 95% CI** | **UL 95% CI** | **p-value** |
| --- | --- | --- | --- | --- |
| Intercept: Dose 0nM vs. DMSO 0.075% | -0.050 | -0.227 | 0.128 | 0.577 |
| Intercept: Dose 1000nM vs. DMSO 0.075% | 0.018 | -0.159 | 0.196 | 0.837 |
| Intercept: Dose 100nM vs. DMSO 0.075% | 0.022 | -0.155 | 0.199 | 0.806 |
| Intercept: Dose 10nM vs. DMSO 0.075% | 0.026 | -0.152 | 0.203 | 0.773 |
| Intercept: Dose 2000nM vs. DMSO 0.075% | 0.025 | -0.153 | 0.202 | 0.782 |
| Intercept: Dose 3000nM vs. DMSO 0.075% | 0.035 | -0.142 | 0.212 | 0.694 |
| Intercept: Dose 300nM vs. DMSO 0.075% | 0.016 | -0.161 | 0.194 | 0.854 |
| Intercept: Dose 30nM vs. DMSO 0.075% | 0.018 | -0.159 | 0.195 | 0.839 |
| Intercept: Dose 3nM vs. DMSO 0.075% | 0.032 | -0.145 | 0.209 | 0.721 |
| Slope: Dose 0nM vs. DMSO 0.075% | -0.001 | -0.002 | 0.001 | 0.535 |
| Slope: Dose 1000nM vs. DMSO 0.075% | -0.004 | -0.006 | -0.002 | <0.001 |
| Slope: Dose 100nM vs. DMSO 0.075% | -0.001 | -0.002 | 0.001 | 0.470 |
| Slope: Dose 10nM vs. DMSO 0.075% | 0.000 | -0.002 | 0.002 | 0.902 |
| Slope: Dose 2000nM vs. DMSO 0.075% | -0.005 | -0.007 | -0.004 | <0.001 |
| Slope: Dose 3000nM vs. DMSO 0.075% | -0.006 | -0.008 | -0.005 | <0.001 |
| Slope: Dose 300nM vs. DMSO 0.075% | -0.001 | -0.003 | 0.001 | 0.177 |
| Slope: Dose 30nM vs. DMSO 0.075% | 0.000 | -0.002 | 0.002 | 0.912 |
| Slope: Dose 3nM vs. DMSO 0.075% | 0.000 | -0.002 | 0.001 | 0.682 |
|  |  |  |  |  |
| **Time trends** | **Estimate** | **LL 95% CI** | **UL 95% CI** | **p-value** |
| Intercept: Dose DMSO0.075% | -1.050 | -1.278 | -0.822 | <0.001 |
| Intercept: Dose 0nM | -1.299 | -1.533 | -1.065 | <0.001 |
| Intercept: Dose 1000nM | -1.120 | -1.354 | -0.886 | <0.001 |
| Intercept: Dose 100nM | -1.022 | -1.256 | -0.788 | <0.001 |
| Intercept: Dose 10nM | -1.043 | -1.277 | -0.809 | <0.001 |
| Intercept: Dose 2000nM | -1.174 | -1.408 | -0.940 | <0.001 |
| Intercept: Dose 3000nM | -1.149 | -1.383 | -0.915 | <0.001 |
| Intercept: Dose 300nM | -1.027 | -1.261 | -0.793 | <0.001 |
| Intercept: Dose 30nM | -1.008 | -1.242 | -0.774 | <0.001 |
| Intercept: Dose 3nM | -1.007 | -1.241 | -0.773 | <0.001 |
| Slope: Dose DMSO0.075% | 0.009 | 0.007 | 0.011 | <0.001 |
| Slope: Dose 0nM | 0.010 | 0.008 | 0.012 | <0.001 |
| Slope: Dose 1000nM | 0.005 | 0.003 | 0.008 | <0.001 |
| Slope: Dose 100nM | 0.008 | 0.006 | 0.011 | <0.001 |
| Slope: Dose 10nM | 0.009 | 0.007 | 0.011 | <0.001 |
| Slope: Dose 2000nM | 0.003 | 0.001 | 0.006 | 0.007 |
| Slope: Dose 3000nM | 0.001 | -0.001 | 0.003 | 0.466 |
| Slope: Dose 300nM | 0.008 | 0.006 | 0.010 | <0.001 |
| Slope: Dose 30nM | 0.009 | 0.007 | 0.011 | <0.001 |
| Slope: Dose 3nM | 0.009 | 0.006 | 0.011 | <0.001 |

**Additional file 6: Table S3.** SUM190 cell proliferation assays: Comparisons from the LME model for log-transformed responses and time trend estimates by CEP-37440 dose level.
