# Supplementary material for: The effects of CEP-37440, an inhibitor of focal adhesion kinase, in vitro and in vivo on inflammatory breast cancer cells
Source: Breast Cancer Res. 2016 Mar 24;18:37. doi: 10.1186/s13058-016-0694-4 (PMC4806466; doi:10.1186/s13058-016-0694-4)
Supplement: Supplementary file 8 — In vivo studies using FC-IBC02 xenograft model: results from the LME model and CEP-37440 treatment comparisons. (DOC 50 kb) [file 13058_2016_694_MOESM8_ESM.doc]

| **Time trends** | **Estimate** | **LL 95% CI** | **UL 95% CI** | **p-value** |
| --- | --- | --- | --- | --- |
| Intercept: Control | 4.38 | 3.26 | 5.50 | <0.001 |
| Intercept: Dose30 | 4.23 | 3.17 | 5.29 | <0.001 |
| Intercept: Dose55 | 4.14 | 3.09 | 5.19 | <0.001 |
| Slope: Control | 0.69 | 0.54 | 0.84 | <0.001 |
| Slope: Dose30 | 0.50 | 0.40 | 0.60 | <0.001 |
| Slope: Dose55 | 0.37 | 0.26 | 0.47 | <0.001 |
| Quadr.coef: Control | -0.04 | -0.06 | -0.03 | <0.001 |
| Quadr.coef: Dose30 | -0.02 | -0.03 | -0.01 | <0.001 |
| Quadr.coef: Dose55 | -0.01 | -0.02 | 0.01 | 0.373 |
| **Treatment comparisons** | | | | |
| **Comparison** | **mean diff.** | **LL 95% CI** | **UL 95% CI** | **p-value** |
| Intercept: Dose30 vs. Control | -0.153 | -0.802 | 0.496 | 0.629 |
| Intercept: Dose55 vs. Control | -0.242 | -0.891 | 0.407 | 0.447 |
| Intercept: Dose55 vs. Dose30 | -0.089 | -0.619 | 0.441 | 0.731 |
| Slope: Dose30 vs. Control | -0.194 | -0.369 | -0.019 | 0.030 |
| Slope: Dose55 vs. Control | -0.325 | -0.508 | -0.143 | 0.001 |
| Slope: Dose55 vs. Dose30 | -0.131 | -0.277 | 0.014 | 0.077 |

**Additional file 8: Table S4.** *In vivo* studies using FC-IBC02 xenograft model: Results from the LME model and CEP-37440 treatment comparisons.
