# Supplementary material for: The effects of CEP-37440, an inhibitor of focal adhesion kinase, in vitro and in vivo on inflammatory breast cancer cells
Source: Breast Cancer Res. 2016 Mar 24;18:37. doi: 10.1186/s13058-016-0694-4 (PMC4806466; doi:10.1186/s13058-016-0694-4)
Supplement: Supplementary file 9 — In vivo studies using SUM149 xenograft model: log-transformed tumor volumes and estimated time trends in each group from the LME model. (DOC 393 kb) [file 13058_2016_694_MOESM9_ESM.doc]

**Additional file 9: Figure S5.** *In vivo* studies using SUM149 xenograft model: Log-transformed tumor volumes and estimated time trends in each group from the LME model. Week, weeks of treatment with CEP-37440.
