# Supplementary material for: The effects of CEP-37440, an inhibitor of focal adhesion kinase, in vitro and in vivo on inflammatory breast cancer cells
Source: Breast Cancer Res. 2016 Mar 24;18:37. doi: 10.1186/s13058-016-0694-4 (PMC4806466; doi:10.1186/s13058-016-0694-4)
Supplement: Supplementary file 10 — In vivo studies using SUM149 xenograft model: results from the LME model and CEP-37440 treatment comparisons. (DOC 56 kb) [file 13058_2016_694_MOESM10_ESM.doc]

| **Time trends** | **Estimate** | **LL 95% CI** | **UL 95% CI** | **p-value** |
| --- | --- | --- | --- | --- |
| Intercept: Dose30 | 0.50 | -1.18 | 2.18 | 0.540 |
| Intercept: Dose55 | 0.89 | -0.87 | 2.66 | 0.302 |
| Intercept: Control | 1.74 | 0.02 | 3.45 | 0.048 |
| Slope: Dose30 | 1.16 | 1.02 | 1.29 | <0.001 |
| Slope: Dose55 | 1.04 | 0.89 | 1.19 | <0.001 |
| Slope: Control | 0.77 | 0.59 | 0.95 | <0.001 |
| Quadr.coef: Dose30 | -0.07 | -0.08 | -0.05 | <0.001 |
| Quadr.coef: Dose55 | -0.05 | -0.07 | -0.03 | <0.001 |
| Quadr.coef: Control | -0.03 | -0.05 | 0.00 | 0.018 |
| **Comparison** | **mean diff.** | **LL 95% CI** | **UL 95% CI** | **p-value** |
| Intercept: Dose30 vs. Control | -1.24 | -1.87 | -0.60 | 0.001 |
| Intercept: Dose55 vs. Control | -0.84 | -1.50 | -0.19 | 0.014 |
| Intercept: Dose55 vs. Dose30 | 0.39 | -0.17 | 0.95 | 0.157 |
| Slope: Dose30 vs. Control | 0.39 | 0.16 | 0.62 | 0.001 |
| Slope: Dose55 vs. Control | 0.27 | 0.03 | 0.51 | 0.025 |
| Slope: Dose55 vs. Dose30 | -0.12 | -0.32 | 0.08 | 0.257 |
| Quadr.coef: Dose30 vs. Control | -0.04 | -0.07 | -0.01 | 0.005 |
| Quadr.coef: Dose55 vs. Control | -0.03 | -0.05 | 0.00 | 0.080 |
| Quadr.coef: Dose55 vs. Dose30 | 0.01 | -0.01 | 0.04 | 0.234 |
| Weight slope | 0.06 | -0.02 | 0.14 | 0.156 |

**Additional file 10: Table S5.** *In vivo* studies using SUM149 xenograft model: Results from the LME model and CEP-37440 treatment comparisons.
