# Supplementary material for: The effects of CEP-37440, an inhibitor of focal adhesion kinase, in vitro and in vivo on inflammatory breast cancer cells
Source: Breast Cancer Res. 2016 Mar 24;18:37. doi: 10.1186/s13058-016-0694-4 (PMC4806466; doi:10.1186/s13058-016-0694-4)
Supplement: Supplementary file 11 — In vivo studies using SUM190 xenograft models. (DOC 39 kb) [file 13058_2016_694_MOESM11_ESM.doc]

I

| **Time trends** | **Estimate** | **LL 95% CI** | **UL 95% CI** | **p-value** |
| --- | --- | --- | --- | --- |
| Intercept: Control | 4.234 | 3.610 | 4.857 | <0.001 |
| Intercept: Dose55 | 3.548 | 2.974 | 4.122 | <0.001 |
| Slope: Control | 0.316 | 0.269 | 0.363 | <0.001 |
| Slope: Dose55 | 0.287 | 0.241 | 0.333 | <0.001 |
| Quadr.coef: Control/Dose55 | -0.015 | -0.018 | -0.012 | <0.001 |
| Cubic.coef: Control/Dose55 | 0.000 | 0.000 | 0.000 | <0.001 |
| **Comparison** | **mean diff.** | **LL 95% CI** | **UL 95% CI** | **p-value** |
| Intercept: Dose 55 vs. Control | -0.685 | -1.439 | 0.068 | 0.069 |
| Slope: Dose 55 vs. Control | -0.029 | -0.046 | -0.013 | 0.001 |
|  |  |  |  |  |
|  | **Estimate** | **LL 95% CI** | **UL 95% CI** | **p-value** |
| **%TGI at day 31 for SUM190**  **(dose 55 vs. control)** | 79.7 | 27.7 | 94.3 | 0.001 |

**Additional file 11: Table S6.** In vivo studies using SUM190 xenograft model: Results from the LME model and CEP-37440 treatment comparisons.
