# Supplementary material for: The effects of CEP-37440, an inhibitor of focal adhesion kinase, in vitro and in vivo on inflammatory breast cancer cells
Source: Breast Cancer Res. 2016 Mar 24;18:37. doi: 10.1186/s13058-016-0694-4 (PMC4806466; doi:10.1186/s13058-016-0694-4)
Supplement: Supplementary file 12 — Supplementary Materials and Methods. Detail description of materials and methods. (DOCX 17 kb) [file 13058_2016_694_MOESM12_ESM.docx]

**Additional file 12**

**Supplementary Material and Methods**

*Cells and Media*

FC-IBC02 and KPL4 were grown in Dulbecco’s modified Eagle’s medium (DMEM) with high glucose, 1.5 g/l NaHCO3, 10% fetal bovine serum (FBS), 1 mM sodium pyruvate, and 1X antibiotic- antimycotics (Gibco, Life Technologies, NY). The IBC cell lines SUM149, SUM190, and MDA-IBC03 were grown in Ham’s F-12 media supplemented with 10% fetal bovine serum (FBS) (Invitrogen, Life Technologies, CA), 5 µg/ml insulin, 1µg/ml hydrocortisone, and 1X antibiotic- antimycotics (Gibco, Life Technologies, NY). The human normal-like breast epithelial cells MCF-10A and MCF-12A were grown in Dulbecco’s modified Eagle’s medium [DMEM/F-12, Gibco, Carlsbad, CA; formula 90–5212 EF: containing DMEM/F12 (1:1) with L-glutamine and phenol red, D-glucose 315 mg/l, sodium pyruvate 55 mg/l] with 5% horse serum, 2.43 g/l sodium bicarbonate, 20 mg/l epidermal growth factor (EGF), 100 mg/l Vibrio cholerae toxin, 10 mg/l insulin, 0.5 mg/l hydrocortisone, 1.05 mM calcium, 100 U/ml penicillin, 100 mg/ml streptomycin, and 0.25 mg/ml amphotericin [22]. The triple negative non-IBC cell lines MDA-MB-231 and MDA-MB-468 were grown in RPMI 1640 media with 2mM L-glutamine, 10% FBS, 100 units/ ml penicillin, and 100 units/ ml streptomycin (Gibco, Life Technologies, NY). Cells were grown in a humidified atmosphere of 5% CO2 at 37 °C.

*Gene Expression Profiling*

FC-IBC02 cells were treated with 1,000 nM CEP-37440 for 48 h. RNA was isolated from the cells using RiboPureTM kit (Life Technologies, NY) and the RNA quality was controlled using the Agilent 2100 Bioanalyzer. Amplification of cDNA was performed from 50 ng RNA using the Ovation Pico WTA-system V2 according NuGen protocol (NuGen Technologies, Inc.). A total of 2.5 μg cDNAs were fragmented and chemically labeled with biotin to generate biotinylated cDNA using FL-Ovation cDNA biotin module (NuGen Technologies, Inc.). Affymetrix gene chips Human gene 1.0 ST array (Affymetrix, Santa Clara, CA) were hybridized with 2.5 μg fragmented and biotin-labeled cDNA in 110 μl of hybridization cocktail. Target denaturation was performed at 99 °C for 2 min and then 45 °C for 5 min, followed by hybridization for 18 h. Arrays were then washed and stained using Gene chip Fluidic Station 450, using Affymetrix GeneChip hybridization wash and stain kit. Chips were scanned on an Affymetrix Gene Chip Scanner 3000, using Command Console Software. Data analyses were performed using GeneSpring software 13.1 (Agilent Technologies, Inc., Santa Clara, CA). The probe set signals were calculated with the Iterative Plier 16 summarization algorithm; baseline to median of all samples was used as baseline option. Data was filtered by percentile and lower cut off was set at 25. The criteria for differentially expressed genes were set at ≥ 2.0-fold changes. Statistical analysis was performed to compare 2 groups using T-test unpaired with p-value less than equal to 0.05. Heat map was generated from differentially expressed gene list. The list of differentially expressed genes was loaded into Ingenuity Pathway Analysis (IPA) 8.0 software (http://www.ingenuity.com) to perform biological network and functional analyses.

*In vivo studies using SCID mice*

The animal experiments were performed in a facility at Thomas Jefferson University that is accredited by the Association for the Assessment and Accreditation of Laboratory Animal Care. Studies were approved by the Institutional Animal Care Committee. The IBC cells were injected in the mammary fat pad (orthotopic) of 35- to 40-day-old female, severe combined immune-deficient (SCID) mice, model number # CB17SCRF (Taconic). For animals harboring FC-IBC02 or SUM149 breast xenografts, the tumor volumes were measured once per week; for animals harboring SUM190 breast tumor xenografts, the tumor volumes were measured every day during weekdays. The animal weights were measured once per week. After 40 days of treatment or when the primary tumor reached a volume of approximately 1 cm^3^, the animals were euthanized by CO_2_ inhalation. Breast tumors and other organs (lungs, heart, liver, spleen, brain, ovaries, kidneys, and lymph nodes) were removed; a piece from each of the breast tumor xenografts was immediately frozen in liquid nitrogen and stored at −80 ˚C in order to measure FAK1 and phospho-FAK1 (Tyr 397). The rest of breast tumor xenografts and other organs were fixed in 10% neutral buffered formalin and paraffin-embedded for histological examination. Paraffin embedded sections of 4-µm thickness were used for hematoxylin/eosin staining.

The CEP-37440 doses were chosen based on preliminary experiments in order to achieve an optimal plasma concentration-response relationship [1, 2].

**References for Additional Information**

1. Discovery of Clinical Candidate CEP-37440: A Selective Inhibitor of Focal Adhesion Kinase (FAK) and Anaplastic Lymphoma Kinase (ALK). Gregory R. Ott, Mangeng Cheng, Keith S. Learn, Jason Wagner, Diane E. Gingrich, Joseph G. Lisko, Matthew Curry, Eugen F. Mesaros, Arup K. Ghose, Matthew R. Quail, Weihua Wan, Lihui Lu, Pawel Dobrzanski, Mark S. Albom, Thelma S. Angeles, Kevin Wells-Knecht, Zeqi Huang, Lisa D. Aimone, Elizabeth Bruckheimer, Nathan Anderson, Jay Friedman, Sandra V. Fernandez, Mark A. Ator, Bruce A. Ruggeri, Bruce D. Dorsey. In preparation.
2. Jacobs M, Ott G, Courvoisier L: Fused bicyclic 2, 4-diaminopyrimidine derivative as a dual ALK and FAK inhibitor (WO2013134353 A1). Patents 2013.
